# Cholesterol Modifies Nutritional Values and Flavor Qualities in Female Swimming Crab (*Portunus trituberculatus*)

**DOI:** 10.1155/2024/7067588

**Published:** 2024-03-07

**Authors:** Tingting Zhu, Min Jin, Hongyu Peng, Wenli Zhao, Yuedong Shen, Shichao Xie, Qicun Zhou

**Affiliations:** ^1^Laboratory of Fish and Shellfish Nutrition, School of Marine Sciences, Ningbo University, Ningbo 315211, China; ^2^Key Laboratory of Aquaculture Biotechnology Ministry of Education, Ningbo University, Ningbo 315211, China; ^3^Key Laboratory of Green Mariculture (Co-construction by Ministry and Province), Ministry of Agriculture and Rural, Ningbo 315211, China

## Abstract

The quality of crustacean aquatic products is affected by feed. Cholesterol (CHO), an essential element for crustacean growth, has been widely supplemented in diet, but its food quality regulation remains unclear. The study aimed to investigate the effects of different dietary CHO levels (0.12%, 1.00%, and 2.50%) on the nutritional value and flavor quality in the edible parts of female swimming crabs (*Portunus trituberculatus*). Results showed that dietary CHO levels significantly increased lipid content in the hepatopancreas and promoted the accumulation of n-3 long-chain polyunsaturated fatty acids (LC-PUFA) in the hepatopancreas and muscle by activating the gene expression related to biosynthesis pathways. However, with dietary CHO levels increased, protein content in muscle decreased significantly. This may be related to dietary CHO supplementation (especially 2.50% CHO level) suppressed amino acid accumulation in the hepatopancreas and muscle by downregulating the target of the rapamycin pathway and upregulating amino acid catabolism-related genes. Moreover, 1.00% CHO treatment had higher relative levels of volatiles, producing grassy, fruity, and fatty odors in muscle, which may be due to the upregulation of the branched-chain amino acid transaminase (*bcat*) expression level. Dietary CHO weakened nucleotide and free amino acid accumulation in hepatopancreas and muscle. Overall, this study suggests that dietary 1.00% CHO level had higher LC-PUFA and pleasing flavor substances in muscle but was not conducive to hepatopancreatic protein and flavor nucleotide deposition of swimming crab.

## 1. Introduction

The quality of aquatic products significantly affects consumer purchasing behavior, ultimately determining aquaculture's commercial value [1]. Numerous studies reported that the quality of aquaculture products depends on the nutritional value and sensory quality of the edible part, both of which are nearly touched on dietary constituents. The nutritional value, including lipid, fatty acids, protein, amino acids, mineral content, and vitamins, while the sensory quality includes color, flavor, texture, and smell/odor [2, 3]. It is commonly accepted that evaluating the nutritional value of seafood is determined by the contents of essential amino acids (EAA) and essential fatty acids (EFA) [4]. Nonvolatile substances, such as flavor nucleotides, free amino acids (FAAs), free sugars, inorganic ions, organic acids, and betaine, interact with taste receptors to form taste [5, 6]. Meat products contain aroma components such as alcohols, aldehydes, ketones, esters, and other volatile substances [7]. Therefore, evaluating the quality of aquatic products should be considered from a multiaspect, including nutrition, taste, and flavor.

Dietary lipids have been shown to significantly impact meat quality, with the sources and levels being crucial factors. For instance, dietary lipids have the ability to change the nutritional and sensory characteristics of fish, such as *Carassius auratus gibelio* [8], *Oreochromis niloticus* [9], and crustaceans, including *Eriocheir sinensis* [7] and *Litopenaeus vannamei* [10]. Cholesterol (CHO), a steroid compound, serves as a precursor for synthesizing important physiological substances and plays a vital role in lipid absorption and transportation in animals [11–13]. However, a special feature of the lipid metabolism of crustaceans is that, unlike vertebrates, they are unable to synthesize CHO *de novo*. This means that crustaceans need to get CHO from the food to ensure the necessary physiological metabolism and growth [14]. Previous studies also indicated that CHO plays a crucial role in endocrinology and lipid metabolism of crustaceans [15, 16]. In *E. sinensis*, dietary CHO may promote the lipid content through downregulating lipolysis and stimulating lipid synthesis, while 0.4% CHO in the diet can increase the estradiol level, which in turn helps with the accumulation of nutrients in the ovary and ultimately leads to the maturation of the ovary and promotes reproductive [17, 18]. In summary, CHO, as an essential lipid nutrient of crustaceans, has a positive potential to regulate meat quality, but the specific regulatory mechanism is still unclear.

The swimming crab (*Portunus trituberculatus*) is a crustacean species with high economic value in Eastern Asia due to its salient nutritional composition and positive flavor attributes [19]. In 2022, the fishing in swimming crab reached 458,297 tons and the aquaculture production reached 109,017 tons [20]. The swimming crab farming and production industries are facing greater challenges due to the increasing demand for healthy, high-quality, safe, delicious, and nutritious crabs [21]. Recently, there has been an increasing amount of research into the flavor qualities of crustaceans, such as mud crab (*Scylla paramamosain*) [22, 23], Chinese mitten crab (*E. sinensis*) [4, 24], *L. vannamei* [25], and swimming crab [26]. However, no data are available on the effect of dietary CHO on the flavor quality of swimming crab. Therefore, a 19-week feeding trial was designed to explore the impacts of dietary CHO on nutritional values and flavor qualities in female swimming crabs, provide strategies for improving food quality in marine crustaceans, and provide new insight into cultivating healthy and nutritious food.

## 2. Materials and Methods

### 2.1. Animal Ethics

The present study adhered to the guidelines set forth by the Animal Research Institute Committee of Ningbo University, China, ensuring that ethical standards were maintained in animal experimentation. Approval from the esteemed Committee of the Animal Research Institute, Ningbo University, China, was obtained to validate the legitimacy of the study.

### 2.2. Experimental Diets

Based on the results of our previous research [27], three different CHO levels diets (0.12%, 1.00%, and 2.50%) were produced, and the formulations and proximate compositions are presented in Table S1. Diets were prepared in the same manner as previously described in our laboratory [27] and contained 46% protein and 8.5% lipid. In order to maintain a balanced lipid content, palmitic acid was supplemented in the experimental diets, resulting in a consistent total amount of 2% in each diet. First, the ground ingredients were mixed, and the progressive enlargement method added micronutrient premixes, including minerals and vitamins. Then, two dietary sizes were made with 2 and 4 mm molds, steam dried to about 5% moisture, and stored at −20°C before the feeding trial. The fatty acid profile of diets is shown in Table S2.

### 2.3. Feeding Trial

The 300 female juvenile swimming crabs were obtained from a hatchery in Hengma nursery farm (Ningbo, China) and acclimated to the experimental conditions in aquaria (100 L, 40 cm × 60 cm × 48 cm). Juvenile crabs were fed a commercial diet containing about 8% lipid and 45% protein during acclimation for 2 weeks. Continuous water purification in a recirculating aquaculture system is performed through filtration treatments. A total of 96 juvenile crabs (1.51 ± 0.08 g) were randomly assigned to 96 single aquaria. Three experimental diets were assigned to four replicates of eight female juvenile crabs each. Juvenile crabs were fed a diet at 8:00 and 18:00. During the experimental period of 19 weeks, environmental conditions were closely monitored, with water temperature ranging from 26.8 to 27.4°C, dissolved oxygen levels maintained between 7.0 and 8.0 mg/L, pH between 7.4 and 7.8, salinity ranging from 23.9 to 26.1 g/L, and ammonia nitrogen levels kept below 0.05 mg/L.

### 2.4. Sample Collection

The crabs were anesthetized on ice for 10 min (to make sure that the crabs were fully anesthetized and unconscious) before sampling. All female crabs were recorded for survival, molting, feed intake, and weight to determine survival, percent weight gain (PWG), feed conversion ratio (FCR), specific growth rate (SGR), and molting ratio. The growth performance is shown in Table S3. Afterward, the hemolymph from four female crabs per replication was collected and stored at 4°C for 24 hr. Subsequently, the hemolymph underwent centrifugation (956x *g*, 10 min, 4°C) to assess biochemical indices (four crabs per replication, *n* = 4). The hepatopancreas and muscle samples were quickly dissected using tweezers from the same four crabs to analyses of proximate composition, contents of CHO and triglyceride (TG), fatty acids, amino acids, FAAs, nucleotides, and gene expression analysis. Additionally, muscle samples from the same four female crabs were promptly placed in 5 mL centrifuge tubes to examine volatile compounds. All the procedures were carried out on ice to ensure accuracy.

The following variables were calculated:

(1) $$\begin{array}{c} \text{Percent weight gain }\left(\text{PWG},\%\right)=\frac{100\times \left(W_{t}-W_{i}\right)}{W_{i}}, \end{array}$$

(2) $$\begin{array}{c} \text{Feed conversion ratio }\left(\text{FCR}\right)=\frac{\text{feed consumed}\left(\text{g},\text{dry weight}\right)}{\text{weight gain}\left(\text{g},\text{wet weight}\right)}, \end{array}$$

(3) $$\begin{array}{c} \text{Specific growth ratio }\left(\text{SGR},\%\;{\text{day}}^{-1}\right)=\frac{100\times \left(\text{Ln }W_{t}-\text{Ln }W_{i}\right)}{t}, \end{array}$$

(4) $$\begin{array}{c} \text{Molting ratio }\left(\text{MR}\right)=\frac{2\times \text{the number of molting}}{\left(N_{t}+N_{i}\right)}, \end{array}$$

where *W*_*t*_ and *W*_*i*_ were the final and initial body weights, respectively; *t* was the duration of experimental days; *N*_*t*_ and *N*_*i*_ were the final and initial crab numbers, respectively.

### 2.5. Dietary CHO Content Analysis

The CHO content in the diets was determined through the gas chromatography (Shimadzu Co., Tokyo, Japan) method, as outlined in a study by Zhu et al. [27]. The 10 mL chloroform was added to 500 mg experiment diets to extract lipids. Subsequently, 1 mL of the lipid solution was dried, added 1 mL of diethyl ether and was analyzed using gas chromatography.

### 2.6. Nutritional Values Analysis

#### 2.6.1. Proximate Composition Analysis

The moisture, ash, lipid, and protein contents in diets, muscle, and hepatopancreas were conducted with standard AOAC [28] methods. Moisture content in the diets, muscle, and hepatopancreas was measured by drying the samples in an oven to constant weight at 105°C. The lipid content was ascertained through petroleum ether extraction using a Soxtec System HT (Tecator Co., Hoganas, Sweden). The crude protein content was measured using the Kjeldahl method, while ash content was determined by incineration in a muffle furnace at 550°C for 8 hr.

#### 2.6.2. The CHO and TG Content Analysis

The CHO (A111-2-1) and TG (A110-2-1) content was measured in the hepatopancreas, muscle, and hemolymph samples using commercial kits from Nanjing Jiancheng Bioengineering Institute.

#### 2.6.3. Fatty Acid Analysis

Fatty acid profile in diets, muscle, and hepatopancreas of female swimming crabs followed the previous method [29]. Briefly, approximately 120 mg of freeze-dried samples were placed in glass tubes. Fatty acid methyl ester (FAME) was prepared with chloroform/methanol (2 : 1 volume ratio) and methanol-sulfuric acid using C23 : 0 as the internal standard. The Agilent Technologies' GC–MS (7890B-5977A, Agilent Technologies CO., California, USA) was used to analyze FAME. The GC–MS detection parameters were meticulously adjusted as follows: the injection temperature was 250°C; the ion source temperature was 230°C. A constant flow rate of 1.0 mL/min of high-purity helium (99.999%) was utilized as the carrier gas. Mass spectra data were acquired in full-scan mode. The fatty acid contents were calculated as follows: (fatty acid/C23 : 0 × correction factor)/(dry matter content × sample weights).

#### 2.6.4. Amino Acid Analysis

Amino acid composition of hepatopancreas and muscle were analyzed based on our previous method [30]. Each sample (approximately 30 mg freeze-dried) was weighed into a 20 mL headspace vial, and 5 mL HCl (6 mol/L) was subsequently added. The headspace vial was sandbathed in at 110°C for 24 hr, then the digested samples were resuspended in 1 mL HCl (0.02 N). Finally, 20 *μ*L of the supernatant sample was measured by an automatic amino acid analyzer (L8900, Hitachi Co., Tokyo, Japan). The sodium citrate buffer system is listed in Table S4, and the amino acid results were expressed as g/100 g dry matter.

### 2.7. Nonvolatile Taste Substances Analysis

#### 2.7.1. FAA Analysis

The FAA of muscle and hepatopancreas was extracted by the trichloroacetic acid (TCA) method, according to the description of Wang et al. [31] and Song et al. [32]. The sample weighed approximately 1.0 g, and 10 mL of 5% TCA was added. After homogenizing at high speed for 20 min, the homogenate was left for 2 hr, centrifuged (10,000x *g*, 4°C, 10 min) to collect 1 mL supernatant and analyzed by liquid chromatography (Ag1100, Agilent Technologies CO., California, USA).

The taste active value (TAV) was expressed by the following formula:

(5) $$\begin{array}{c} \text{TAV}=\frac{\text{the compounds' concentration}}{\text{the threshold value}}. \end{array}$$

Flavor contributors were defined as compounds with TAV greater than 1.

#### 2.7.2. Nucleotides Analysis

The nucleotide content of muscle and hepatopancreas was measured according to the description of Tao et al. [33]. The extracted solutions were determined by high-performance liquid chromatography (HPLC, 2,695e; Waters Ltd., Milford, MA). The HPLC detection parameters are as follows: chromatographic column, Diamonsil C18 (4.6 mm × 250 mm); column temperature, 30°C; UV detector wavelength, 254 nm; injection volume, 5 *μ*L. The eluent compositions, gradient, and HPLC program are listed in Table S5.

#### 2.7.3. Equivalent Umami Concentration (EUC) Analysis

Nucleotides synergize with umami amino acids, which can increase umami flavor several times and are represented by EUC (the concentration of monosodium glutamate (MSG) in a 100 g sample). The equation expressed the EUC as follows:

(6) $$\begin{array}{c} \text{EUC}=\sum \text{AiBi}+1,218\left(\sum \text{AiBi}\right)\left(\sum \text{AjBj}\right), \end{array}$$

where EUC was g MSG/100 g sample; Ai, umami amino acid (Asp, Glu) concentration (g/100 g); Bi, amino acid conversion coefficient of umami flavor (Asp, 0.077; Glu, 1); Aj, 5'-nucleotide concentration (g/100 g); Bj, 5'-nucleotide conversion coefficient (IMP: 0.18; AMP, 0.18; GMP, 2.3); 1,218, the synergy constant.

### 2.8. Volatile Compounds Analysis

Identification of volatile compounds in muscles as described by Luo et al. [34]. First, approximately 4 g of muscle was weighed into a 20 mL headspace vial using an electronic balance. Added 3 *µ*L 2,4,6-trimethylpyridine (TMP, 100 mg/L, internal standard). To facilitate the extraction process, 5 mL of saturated NaCl solution was introduced, after which the mixture was thoroughly combined in a temperature-controlled water bath set at 60°C for 30 min. The extraction of volatile substances from the muscle samples was accomplished through the application of GC–MS. The GC–MS detection parameters were meticulously adjusted as follows: column temperature, 35–200°C; the column, consisting of dimensions 60 m × 0.32 mm × 0.25 *µ*m, was utilized; a highly pure helium carrier gas with a purity of 99.999% was employed; injector temperature, 210°C; a 70 eV ionization energy was employed for ionization; the flow rate was 2.25 mL/min and the ion source temperature was attentively controlled at 220°C.

The data generated by the mass spectrometry was collected and subsequently processed using the Agilent MassHunter workstation (Agilent Technologies, California, USA). The volatile compounds were identified by the NIST14.L mass spectral library or standard compounds, with a score matching greater than 85% of the acceptance criterion.

To quantitatively determine each volatile compound's relative concentration (ng/g), the peak area ratio of each compound to the internal standard was calculated.

### 2.9. Gene Expression

The RNA extraction and qPCR were determined as described by Wang et al. [35]. The muscle and hepatopancreas samples were used for synthesizing cDNA using a commercial kit (Vazyme, China). The primer sequences are listed in Table S6. The qPCR was run in Lightcycler 96 (Roche Co., Basel, Switzerland), and the fluorescence results were obtained. The 2^−*ΔΔ*Ct^ method was used to calculate the expression levels of genes [36]. In order to further explore the regulatory effects of CHO on fatty acids, amino acids and volatile substances, the genes related to fatty acid synthesis (fatty acyl desaturase 2 (*fad2*), elongase (*elovl*), and elongase 4 (*elovl4*)), amino acid metabolism (aminotransferase (*ast*), alanine aminotransferase 1 (*alt1*), L-threonine 3-dehydrogenase (*tdh*), ribosomal protein S6 (*s6*), ribosomal protein S6 kinase1 (*s6k1*), target of rapamycin (*tor*), eukaryotic initiation factor 4E-binding protein-1 (*4e-bp1*), protein kinases B (*akt*), eukaryotic translation initiation factor 4E-1A (*eif4e1a*), eukaryotic translation initiation factor 4E-3 (*eif4e3*), eukaryotic translation initiation factor 4E-2 (*eif4e2*)) and volatile substances metabolism (lipoxygenase (*lox*), alcohol dehydrogenase (*adh*), branched-chain amino acid transaminase (*bcat*)) were analyzed.

### 2.10. Calculations and Statistical Analysis

Data were displayed as mean ± SEM and variance homogeneity was assessed by Levene's test before one-way analysis of variance (ANOVA). Tukey's multiple range test was utilized with a significance threshold set at *P* < 0.05. All statistics were performed using the SPSS package (version 19.0). The correlation analysis, heat map, hierarchical cluster analysis (HCA) of multidimensional correlation analysis, and principal component analysis (PCA) were analyzed by the online program ChiPlot (https://www.chiplot.online/).

## 3. Results

### 3.1. Growth Performance

The effects of dietary CHO levels on growth performance and feed utilization of female swimming crabs are shown in Table S3. There was no significant difference in survival of female swimming crabs (*P* > 0.05). Moreover, 1.00% and 2.50% treatments showed significantly higher FBW, PWG, and SGR compared to 0.12% treatment (*P* < 0.05).

### 3.2. Nutrition Values

#### 3.2.1. Proximate Composition

**Figure 1:**
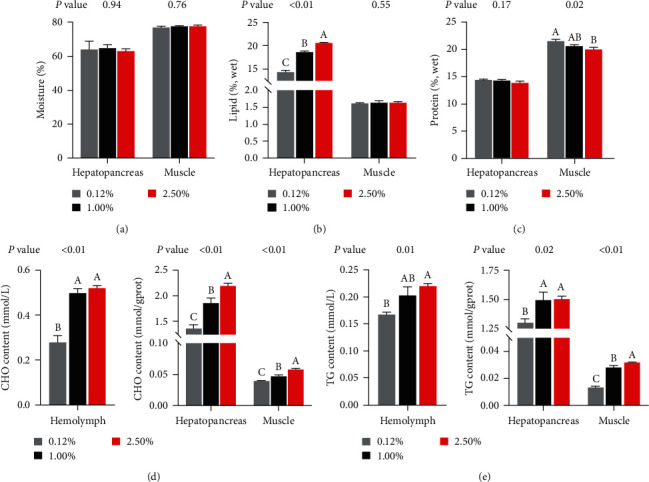
Effects of dietary CHO levels on the proximate composition, CHO, and TG contents in tissues of female swimming crab. Data are represented as the mean and SEM of four replicates. Columns represent means with bars indicating standard error. Values within the same row with different superscripts are significantly different *P* < 0.05. (a–c) Proximate composition in hepatopancreas and muscle; (d) CHO content in hemolymph, hepatopancreas, and muscle; (e) TG content in hemolymph, hepatopancreas, and muscle. CHO, cholesterol; TG, triglyceride.

The moisture, lipid, and protein contents in the hepatopancreas and muscle of female swimming crabs are recorded in Figure 1(a)–1(c). No significant differences were found among all treatments in terms of hepatopancreatic protein and moisture contents, as well as muscular lipid and moisture contents (*P* > 0.05). With dietary CHO levels increased, the lipid content in the hepatopancreas increased significantly, and the protein content in muscle decreased significantly (*P* < 0.05).

#### 3.2.2. Contents of CHO and TG

The contents of CHO and TG in hemolymph, hepatopancreas, and muscle of female swimming crabs are shown in Figures 1(d) and 1(e). In general, the CHO and TG contents in these three tissues increased significantly with increased dietary CHO levels (*P* < 0.05). Specifically, CHO content in hemolymph and TG content in hepatopancreas in 1.00% and 2.50% treatments were significantly higher than in 0.12% CHO treatment (*P* < 0.05), while those results were no significant influences between 1.00% and 2.50% treatments (*P* > 0.05).

#### 3.2.3. Fatty Acid Metabolism

**Figure 2:**
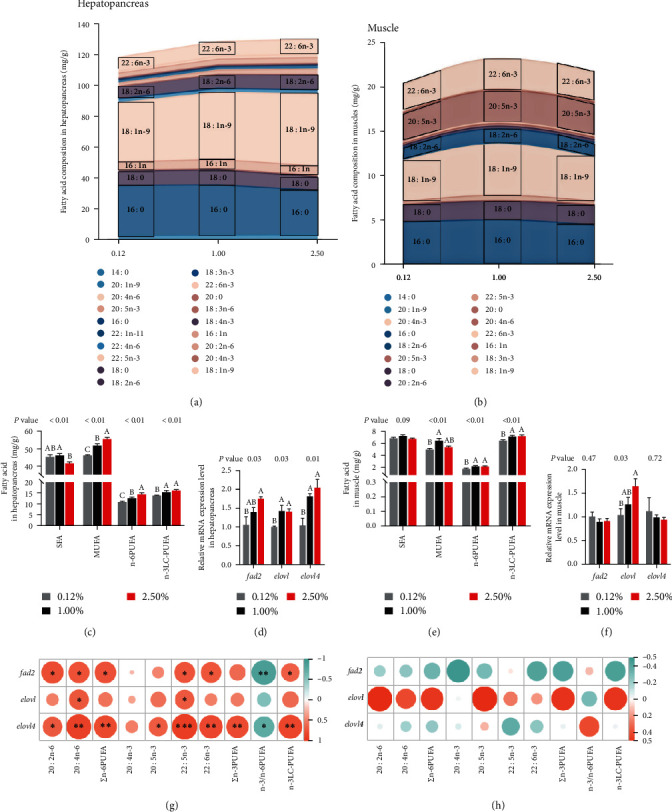
Effects of dietary cholesterol levels on fatty acid metabolism of female swimming crab. (a and b) Stacked area chart of fatty acid profiles in hepatopancreas and muscle; (c and e) SFA, MUFA, n-6PUFA, and n-3 LC-PUFA contents in hepatopancreas and muscle (mg/g dry matter); (d and f) relative expression of genes involved in LC-PUFA biosynthesis in hepatopancreas and muscle of female swimming crab; (g and h) heat map of multidimensional correlation between fatty acid contents and LC-PUFA biosynthesis-related gene expression levels in hepatopancreas and muscle. The existence of “ ^*∗*^” indicates a significant difference between the two comparisons. Data are reported as the mean and SEM of four replicates. Columns represent means with bars indicating standard error. Values within the same row with different superscripts are significantly different *P* < 0.05. SFA, saturated fatty acids; MUFA, monounsaturated fatty acids; n-6 PUFA, n-6 polyunsaturated fatty acids; n-3 LC-PUFA, n-3 long-chain polyunsaturated fatty acids; *fad2*, fatty acyl desaturase 2; *elovl*, elongase; *elovl4*, elongase 4.

The major fatty acid profiles in the hepatopancreas and muscle of female swimming crabs are exhibited in Figures 2(a)–2(c) and 2(e). Besides, the complete fatty acid profiles in hepatopancreas and muscle are listed in Tables S7 and S8, respectively. The highest content of all detected hepatopancreas and muscle fatty acids was 18 : 1n-9, followed by 16 : 0 (Figures 2(a) and 2(b)). Detailly, 2.50% treatment significantly decreased the saturated fatty acids (SFA) content and n-3/n-6 polyunsaturated fatty acids (n-3/n-6 PUFA) ratio in hepatopancreas than the 1.00% CHO treatment (*P* < 0.05). On the contrary, the content of docosahexaenoic acid (DHA), eicosapentaenoic acid (EPA), monounsaturated fatty acids (MUFA), arachidonic acid (ARA), n-6 PUFA, n-3 PUFA, n-3 LC-PUFA and n-6 LC-PUFA in hepatopancreas were significantly increased in 2.50% CHO treatment compared to 0.12% CHO treatment (*P* < 0.05). No statistical differences in ARA, DHA, EPA, n-3 PUFA, and n-3 LC-PUFA contents in hepatopancreas were recorded between 1.00% and 2.50% treatments (*P* > 0.05; Figure 2(c) and Table S7). A similar result was also found in muscle; the contents of ARA, DHA, n-6PUFA, n-3PUFA, n-3 LC-PUFA, and n-6 LC-PUFA in 1.00% and 2.50% CHO treatments were significantly higher compared to 0.12% treatment (*P* < 0.05; Figure 2(e) and Table S8).

The gene expression of hepatopancreas and muscle of female swimming crabs involved in LC-PUFA biosynthesis are recorded in Figures 2(d) and 2(f), respectively. The mRNA expression level of *fad2* in hepatopancreas was significantly upregulated with increased dietary CHO levels (*P* < 0.05). Besides, the higher expression of *elovl* and *elovl4* in hepatopancreas were shown in crabs fed diet with 1.00% and 2.50% CHO levels (*P* < 0.05; Figure 2(d)). In muscle, the expression of *elovl* was statistically upregulated with increased dietary CHO levels (*P* < 0.05). Nevertheless, no remarkable influences were observed in *fad2* and *elovl4* mRNA expression levels in muscle (*P* > 0.05; Figure 2(f)). In addition, the correlation between LC-PUFA biosynthesis-related genes expression level and LC-PUFA content in hepatopancreas was significant (Figure 2(g)). However, the correlation was not significant in muscle (Figure 2(h)).

#### 3.2.4. Amino Acid Metabolism

**Figure 3:**
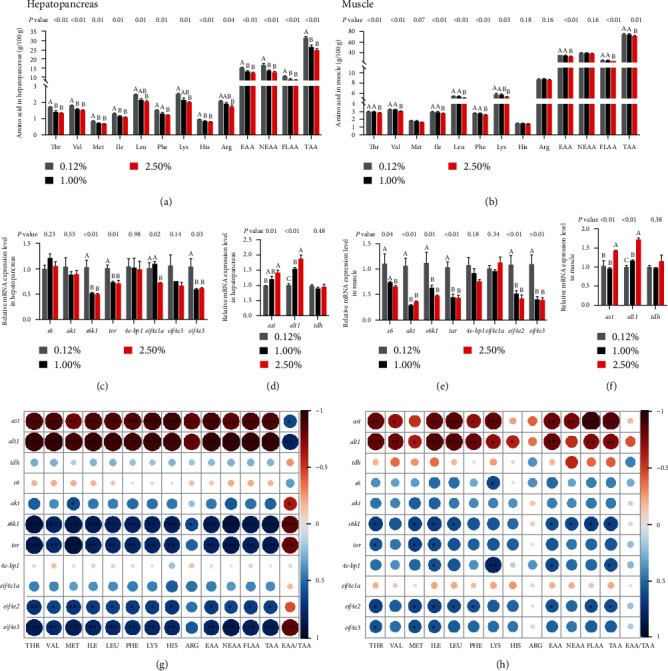
Effects of dietary cholesterol levels on amino acid metabolism of female swimming crab. (a and b) Total amino acid contents (g/100 g dry matter) in hepatopancreas and muscle; (c and e) relative expression of genes involved in TOR pathway in hepatopancreas and muscle of female swimming crab; (d and f) relative expression of genes of aspartate aminotransferase (*ast*), alanine aminotransferase 1 (*alt1*), and L-threonine 3-dehydrogenase (*tdh*) in hepatopancreas and muscle of female swimming crab; (g and h) heat map of multidimensional correlation between amino acid contents and amino acid metabolism-related gene expression levels in hepatopancreas and muscle. The existence of “ ^*∗*^” indicates a significant difference between the two comparisons. Data are reported as the mean and SEM of four replicates. Columns represent means with bars indicating standard error. Values within the same row with different superscripts are significantly different *P* < 0.05. EAA, essential amino acids; NEAA, nonessential amino acids; FLAA, flavor amino acids, including aspartic acid, glutamic acid, glycine, and alanine; TAA, total amino acids. *s6*, ribosomal protein S6; *akt*, protein kinases B; *s6k1*, ribosomal protein S6 kinase1; *tor*, target of rapamycin; *4e-bp1*, eukaryotic initiation factor 4E-binding protein-1; *eif4e1a*, eukaryotic translation initiation factor *4E-1A*; *eif4e2*, eukaryotic translation initiation factor 4E-2; *eif4e3*, eukaryotic translation initiation factor 4E-3.

The major amino acid compositions in the hepatopancreas and muscle of female swimming crabs are recorded in Figures 3(a) and 3(b), respectively. Moreover, the complete amino acid compositions in the hepatopancreas and muscle are presented in Tables S9 and S10, respectively. Obviously, most amino acids in the hepatopancreas and muscle of swimming crabs significantly decreased with increased dietary CHO levels (*P* < 0.05). Specifically, in the hepatopancreas, the contents of threonine (Thr), methionine (Met), histidine (His), valine (Val), isoleucine (Ile), EAA, nonessential amino acids (NEAA), flavor amino acids (FLAA), and total amino acids (TAA) in 0.12% CHO treatment were significantly higher than other CHO treatments (Figure 3(a)). However, in muscle, no difference in contents of Thr, Val, Ile, leucine (Leu), phenylalanine (Phe), EAA, FLAA, and TAA were found between 0.12% and 1.00% treatment (*P* > 0.05), which both significantly higher compared to 2.50% treatment (*P* < 0.05; Figure 3(b)).

In hepatopancreas, no significant influence was observed in the amino acids metabolism-related genes, including mTOR pathway genes like *s6*, *akt*, *4e - bp1*, as well as *eif4e2* expression levels among all treatments (*P* > 0.05; Figures 3(c) and 3(d). Dietary CHO levels significantly upregulated the mRNA level of *alt1* and *astP* < 0.05. Moreover, the expression level of *tor*, *s6k1*, and *eif4e3* in 1.00% and 2.50% CHO treatments were significantly lower than in 0.12% CHO treatment (*P* < 0.05). The gene expression levels of *s6k1*, *tor*, *eif4a2*, and *eif4e3* were positively correlated with most of the amino acid content in hepatopancreas. However, the *ast* and *alt1* mRNA levels were negatively correlated with most of the amino acid content (Figure 3(g)).

Likewise, in muscle, similar results were also presented in *akt*, *s6k1*, *tor*, *eif4e2*, and *eif4e3* expression levels, which showed that the expression level of those genes remarkably downregulated by 1.00% and 2.50% CHO treatments than 0.12% CHO treatment (*P* < 0.05; Figures 3(e) and 3(f). Although, no significant influences were recorded in the expression level of *tdh*, *4e-bp1*, and *eif4e1aP* > 0.05. 2.50% CHO treatment significantly upregulated *ast* and *alt1* gene expression levels than 0.12% CHO treatment (*P* < 0.05). Except for *ast* and *alt1*, the gene expression levels of *s6k1*, *tor*, *eif4a2*, and *eif4e3* were positively correlated with most amino acid content in muscle (Figure 3(h)).

### 3.3. Nonvolatile Taste Substances

#### 3.3.1. Free Amino Acid

**Figure 4:**
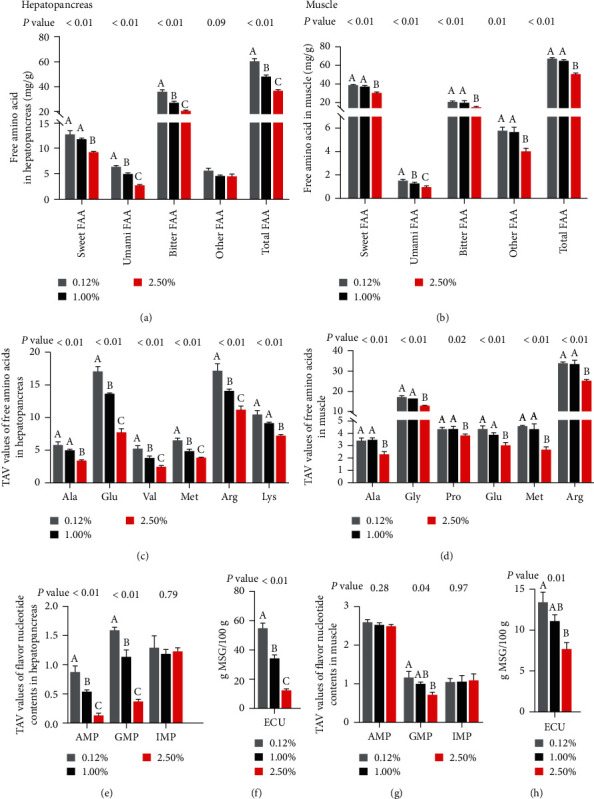
Effects of dietary cholesterol levels on the taste compounds of female swimming crab. (a and b) Taste FAA contents of hepatopancreas and muscle (mg/g dry matter); (c and d) TAV values (top 6) of free amino acids (FAAs) of hepatopancreas and muscle; (e and g) TAV values of flavor nucleotide contents of hepatopancreas and muscle; (f and h) EUC (g MSG/100 g) of hepatopancreas and muscle. Data are reported as the mean and SEM of four replicates. Columns represent means with bars indicating standard error. Values within the same row with different superscripts are significantly different *P* < 0.05.

The bitter FAA, sweet FAA, umami FAA, and total FAA contents in the hepatopancreas of female swimming crabs significantly decreased with increased dietary CHO levels (*P* < 0.05; Figure 4(a) and Table S11). In addition, the TAV values of 11 FAA in hepatopancreas were greater than 1, and these TAV values significantly decreased with the increase of dietary CHO level (*P* < 0.05; Figure 4(c) and Table S12). However, six FAA in muscles with TAV > 1, including alanine (Ala), glycine (Gly), proline (Pro), glutamic acid (Glu), Met, and Arg (Figure 4(d) and Table S13). About 2.50% CHO treatment significantly decreased the sweet FAA, umami FAA, bitter FAA, and total FAA contents and TAV values of six FAA in muscle than other treatments (*P* < 0.05; Figure 4(b) and Table S14).

#### 3.3.2. Flavor Nucleotides

The supplementation of CHO in the diet reduced the contents of AMP and GMP, and the TAV value of AMP and GMP in hepatopancreas (*P* < 0.05; Figure 4(e) and Table S15). Under the different CHO treatments, only the TAV value of IMP in hepatopancreas was greater than 1, although no significant differences were found (*P* > 0.05). In muscle, dietary CHO supplementation significantly reduced the TAV value of GMP (*P* < 0.05; Figure 4(g) and Table S16). No significant influences among treatments in the TAV value of AMP and IMP in muscle (*P* > 0.05).

#### 3.3.3. EUC

The umami intensity of 100 g female swimming crabs in hepatopancreas and muscle were equivalent to 12.49–55.07 g MSG and 7.78 to 13.50 g MSG, respectively (Figure 4(f) and 4(h)). The EUC value of hepatopancreas and muscle in 0.12% CHO treatment was significantly higher than in 2.50% CHO treatment (*P* < 0.05).

### 3.4. Volatile Substances

**Figure 5:**
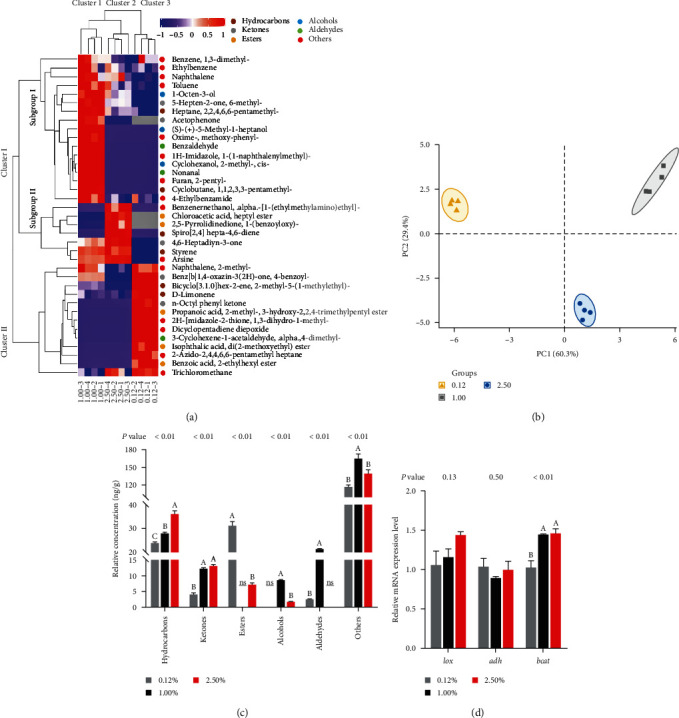
Effects of dietary cholesterol levels on volatile compounds in the muscle of female swimming crab. (a) Hierarchical cluster analysis (HCA) and heat map visualization of samples and volatile compounds of muscle; (b) principal component analysis (PCA) of all volatile compounds.; (c) relative concentration (ng/g) of different kinds of volatile compounds of muscle; (d) relative expression of genes of aspartate lipoxygenase (*lox*), alcohol dehydrogenase (*adh*), and branched-chain amino acid transaminase (*bcat*) in the muscle of female swimming crab. Data are reported as the mean and SEM of four replicates. Columns represent means with bars indicating standard error. Values within the same row with different superscripts are significantly different *P* < 0.05.

The relative quantification (ng/g) of volatile compounds in muscle is shown in Table S17. According to the dendrogram (Figure 5(a)), the diets of each group were divided into 0.12% CHO group (group 3), 1.00% CHO group (group 1), and 2.50% CHO group (group 2). Thirty-seven volatile compounds, including six hydrocarbons, five ketones, five esters, three alcohols, three aldehydes, and 15 other volatile compounds. Additionally, a total of 21, 25, and 18 volatile compounds were identified in muscle in crabs fed diet with 0.12%, 1.00%, and 2.50% CHO levels, respectively. In detail, total aldehydes include 3-cyclohexene-1-acetaldehyde, alpha, 4-dimethyl-, benzaldehyde and nonanal. Interestingly, benzaldehyde and nonanal were only detected in 1.00% CHO treatment. Alcohols included mainly cyclohexanol, 2-methyl-, cis-, 1-octen-3-ol, and (S)-(+)-5-methyl-1-heptanol, which were all highest found in 1.00% CHO treatment. However, alcohol was not measured in 0.12% CHO treatment. PCA of the 37 identified volatile compounds in muscle exhibited that PC 1 accounted for 60.3% of the total variance and PC 2 for 29.4% of the total variance, respectively (Figure 5(b)). The profiles of the volatile compounds were divided into three clusters: 0.12%, 1.00%, and 2.50% treatment. Besides, 2.50% CHO treatment exhibited higher contents of hydrocarbons than other treatments (Figure 5(c)). Esters, aldehydes, and alcohols in muscle were not detected in 1.00%, 0.12%, and 2.50% CHO treatment, respectively (Figure 5(c)).

Dietary CHO levels influence the genes expression involved in volatile substances metabolism (Figure 5(d)). The expression level of *bcat* in muscle in 1.00% and 2.50% CHO treatment were significantly higher compared to 0.12% CHO treatment (*P* < 0.05), whereas no significant influence in *lox* and *adh* expression level of muscle were found among three treatments (*P* > 0.05).

## 4. Discussion

The hepatopancreas and muscle represent two primary edible tissues of swimming crabs, and their nutritional quality directly affects market prices [19]. The proximate composition is a vital indicator for the assessment of the nutritional value of the crab edible tissues [37]. In this study, dietary CHO levels increased lipid content in the hepatopancreas; however, no significant influences in lipid contents of muscle among all treatments. This result was different with a previous study in which feeding a 0.40% CHO diet increased muscular lipid content in white shrimp (*L. vannamei*) [38]. Moreover, Han et al. [15] revealed that *P. trituberculatus* fed a diet with 0.2%–1.4% CHO did not change the proximate composition of muscle and hepatopancreas, except for muscular lipid content. The main reasons for inconsistent results might be attributed to species, dietary formulations, and associated experimental conditions.

The lipids in the hepatopancreas of crustaceans can provide energy, supply the EFA to maintain the integrity of cell membranes and provide CHO for the synthesis of ecdysone [39]. Previously, swimming crabs fed a diet with 1.0%–1.4% CHO increased the CHO contents in the whole body [15]. With the dietary CHO levels rising from 0% to 1.0%, the hepatopancreas and intestinal CHO contents of crayfish (*Procambarus clarkii*) were significantly increased [16]. A similar result was also found in this study. The CHO content in hemolymph, hepatopancreas, and muscle was increased with the increased dietary CHO levels. Besides, compared to muscle, CHO tended to accumulate in the hepatopancreas. Excessive CHO accumulation may bring adverse effects on health. Therefore, dietary CHO supplementation in crustaceans needs to be controlled within the appropriate range, such as *L. vannamei*-fed diet contained 0.92% CHO could satisfy the CHO requirement [40], and oriental river prawn (*Macrobrachium nipponense*) fed a diet supplemented with 0.9% CHO had the best growth performance [41]. In addition, TG, as one of the lipid classes, plays a vital role in organ function, cellular biology, and lipid metabolism [42]. In this study, TG content in hemolymph, muscle, and hepatopancreas was increased by dietary CHO supplementation, similar to the result of lipid content, which further indicated dietary CHO could promote lipid accumulation in female swimming crabs.

Fatty acids play diverse roles by providing essential compounds and energy, enhancing flavor, boosting disease resistance, and promoting vitality and health. Notably, they significantly reduce the risk of cancer and coronary heart disease [19, 43–46]. Moreover, fatty acid profiles in tissues reflect nutritional quality, and our previous studies indicated that diet strongly influences the fatty acid profiles of swimming crabs [47]. In this study, dietary CHO levels increased the contents of ARA, EPA, n-3 LC-PUFA, and n-6 LC-PUFA in hepatopancreas or ARA, DHA, n-3 LC-PUFA, and n-6 LC-PUFA in muscle. This was in accordance with a previous study in black seabream (*Acanthopagrus schlegelii*) fed diet with 1.0% CHO [48]. Different with crustaceans, the need for exogenous CHO is still being debated, as bony fish have the ability to synthesize CHO [37]. Therefore, the mechanism of CHO promoting LC-PUFA synthesis needs further study. Interestingly, we noticed that hepatopancreatic n-3 LC-PUFA contents were significantly higher than those in the muscle, confirming that hepatopancreas is more nutritious than the muscle from the perspective of fatty acid nutrition. Furthermore, the present data showed that 2.50% CHO treatment had a remarkably higher ratio of n-3 PUFA/n-6 PUFA, n-6 PUFA, and n-6 LC-PUFA content compared to 0.12% and 1.00% CHO treatments. Some kinds of n-6 PUFA may play the pro-inflammatory mediators by oxidation of low-density lipoprotein (LDL) and the conversion of 18 : 2n-6 to arachidonic acid, which adversely affects health [44]. Besides, ARA is the main n-6 LC-PUFA, and excessive intake of ARA in aquatic animals can cause apoptosis and oxidative stress, resulting in inflammatory responses [49]. Consequently, female swimming crabs fed the diet with 1.00% CHO is more beneficial for consumers due to the high level of n-3 LC-PUFA and low level of n-6 LC-PUFA in the edible portions.

Except for diet, an endogenous supply of LC-PUFAs can also be provided through their biosynthesis. More specifically, C18 PUFA can utilize the activities of two enzymes, elongating very long-chain fatty acid (Elovl) and front-end desaturase (Fad), to synthesize LC-PUFA [50]. To delve deeper into the role of CHO in the promotion of fatty acid deposition, the *fads2*, *elovl*, and *elovl4* mRNA expression levels were analyzed. In the present study, 1.00% and 2.50% CHO treatments remarkablely upregulated the mRNA expression levels of *elovl* and *elovl4* in hepatopancreas than 0.12% CHO treatment, which are in agreement with the effect that black seabream fed 1.00% CHO diet activate the expression levels of *elovl5* and *fads2* [48]. Besides, those data have shown that CHO significantly increased expression of *fads2* in hepatopancreas and *elovl* in muscle. These results indicated that 1.00% CHO promotes the biosynthesis of LC-PUFA by activating the expression levels of *fad2*, *elovl*, and *elovl4*.

Except of lipids, the supply of high-quality protein is also a vital reflection of the quality of aquatic products. Proteins have a crucial function in the structure and metabolism of living organisms [19]. Amino acids, the basic unit of protein, are organic compounds that have a close relationship with the life activities of organisms and are among the indispensable nutrients in the body [51]. Protein comprises about 20 amino acids, including nine kinds of EAA and other NEAA [24]. EAA cannot be synthesized or can be insufficiently synthesized by an organism but can be obtained through food [52]. In this study, the contents of EAA, NEAA, FLAA, and TAA in hepatopancreas in 1.00% and 2.50% CHO treatments were lower than 0.12% CHO treatment. Similar results were also found in muscle; 2.50% CHO treatment had a significantly lower content of EAA, NEAA, FLAA, and TAA compared with other treatments. These results demonstrated that tissue amino acid deposition is inhibited by high dietary CHO levels, particularly in hepatopancreas, which are more susceptible to dietary CHO levels. In addition, the EAA/TAA ratio is a crucial reference index used to assess the nutritional value of amino acids in aquatic products, and the FAO/WHO/UNU [53] suggests that the ideal EAA/TAA is approximately 0.4, with higher ratios (>0.4) more beneficial to human health. In this study, the ratio of EAA/TAA in hepatopancreas and muscle ranged from 0.48 to 0.50 (Table S9) or 0.46 to 0.47 (Table S10), respectively, which were consistent with a previous study in swimming crabs [21]. Moreover, contents of EAA, NEAA, FLAA, and TAA in muscle were approximately 2.4 folds of those in hepatopancreas. In short, the amino acid results revealed that: (1) the amino acids are more accessible to deposit in muscle than in hepatopancreas; (2) female swimming crabs have good quality in amino acid composition in muscle and hepatopancreas, where all these two tissues could be regarded as being a good source of protein; (3) dietary high CHO level suppresses the synthesis of amino acids, which is not conducive to supplying high nutritional quality protein for human consumption.

To further investigate the impact of CHO on inhibiting amino acid deposition, the mRNA expression levels of amino acid metabolism were analyzed. The aspartate aminotransferase (AST) and alanine aminotransferase (ALT) are the two critical enzymes in amino acid catabolism by transamination of amino acid to precursors of the tricarboxylic acid cycle [35]. Besides, L-threonine 3-dehydrogenase (TDH) is another key enzyme involved in amino acid metabolism by catabolizing threonine [54]. Dietary CHO levels significantly upregulated the expression levels of *alt1* and *ast* both in hepatopancreas and muscle. Those results suggested that dietary CHO levels decreased amino acid content in hepatopancreas and muscle by upregulating expression levels of amino acid catabolism-related genes. Moreover, nutrients could stimulate protein breakdown by inhibiting the TOR pathway and cause a series of upstream (*akt*) or downstream gene (*s6*, *s6k1*, *4e - bp1*, *eif4e2*, *eif4e1a*, and *eif4e3*) responses [35]. The present results revealed that dietary CHO levels could inhibit the expression of *akt* in muscle, which leads to the reduction expression of *tor*. Additionally, the mRNA expression levels of *s6k1*, *s6*, *eif4e2*, and *eif4e3* observed the same trend as *tor* in muscle. Similar results were also found in hepatopancreas. These results are similar to a study on Nile tilapia (*O. niloticus*) [55], further suggesting that CHO intake stimulates protein catabolism as a major energy source, changing nutrient metabolism patterns.

The taste is a crucial attribute affecting consumers' purchasing behavior. FAA directly affects the taste and indirectly participates in flavor development [56]. In addition, a previous study reported that several amino acids (like arginine (bitter), threonine (sweet), and aspartic acid (umami) were important taste compounds in crustaceans [24]. In this study, dietary CHO levels reduced the sweet FAA, umami FAA, bitter FAA, and total FAA contents in the hepatopancreas. However, only high dietary CHO levels (2.50%) reduced those FAA contents in muscle, except for umami FAA. Those findings confirmed that dietary CHO had less effect on the muscles than on the hepatopancreas, perhaps because the muscles of crabs are the most structurally sound of all edible parts [31]. The hepatopancreas is a greasy and delicious edible part that plays a vital role in regulating crab's physiological metabolism. The hepatopancreas is the organ most susceptible to the influence of feeds and environmental factors [31]. The TAV is the ratio of an individual taste compound to its corresponding taste recognition threshold used to evaluate taste-active compounds. FAAs with TAV > 1 were active on the overall taste, and TAV < 1 was considered to have no obvious contribution to taste [23]. Total eleven FAA in hepatopancreas with TAV > 1; however, only six FAA in muscle with TAV > 1. As two of umami FAA, Asp was present in minor amounts in the hepatopancreas and muscle. The Glu content was higher in the hepatopancreas than in the muscle, and their TAV was also much higher. The results suggested that the umami taste of hepatopancreas is more delicious than muscle. Overall, the present results revealed that dietary CHO levels, especially high CHO levels (2.5%), have influenced amino acid catabolism, which may result in a weaker umami taste.

In addition to FAAs, flavor nucleotides are also an important part of taste formation, which include GMP, AMP, and IMP. AMP is the most abundant nucleotide in all tissues of crabs [32]. In this study, IMP content in muscle and hepatopancreas was higher than GMP. Similar observations, albeit with slightly different values, were recorded by Song et al. [32] and Luo et al. [34] in their analysis of nucleotides in the muscles of swimming crabs and mud crabs. Moreover, dietary CHO levels reduced the hepatopancreatic AMP, GMP, and muscular GMP content. Furthermore, dietary CHO levels also reduced the EUC in the hepatopancreas and muscle, although EUC was higher in the hepatopancreas than in the muscle. These indicated that dietary CHO remarkably reduced the umami intensity of hepatopancreas and muscle in female swimming crabs. Based on the comprehensive analysis, these findings further confirmed that dietary CHO levels might regulate nucleotide and FAA metabolism, leading to decreased flavor, as described above.

Odor characteristics are an important factor in determining consumers' purchase of aquatic products, which is mainly determined by volatile flavor substances. Volatile substances, including aldehydes, alcohols, furans, ketones, and lipids, were the main aroma components of crabs [57]. Compared to other volatile compounds in aquatic products, aldehydes are considered the primary flavor source due to their higher content and lower threshold [58]. Most aldehydes in the muscles of swimming crabs have fruity and fatty aromas. For example, nonanal, a major odor of the Chinese mitten crab, has a meaty or grassy odor [31]. In this study, the total aldehyde content in muscle in 1.00% CHO treatment was the highest among all treatments; however, these were not detected in 2.50% CHO treatment. Furthermore, nonanal, a pleasant odor compound, in 1.00% CHO treatment was also significantly higher than 0.12% CHO treatment. These results suggested that crabs fed a 1.00% CHO diet have a more pleasant odor in terms of beneficial aldehydes.

Most esters, alkanes, and alcohols make a small contribution to overall flavor due to the high threshold [23]. Of note among alcohols, 1-octen-3-ol is the primary volatile odor active alcohol in many crustaceans and contributes to the grassy aroma of crabs [59]. The present data showed that 1-octen-3-ol content in 1.00% CHO treatment was significantly higher than in 2.50% CHO treatment, and its content was not measured in 0.12% CHO treatment. Other volatile substances, such as furans, have common aromas of nuts, meat, and caramel [60]. In the present study, furan, 2-pentyl- only detected in 1.00% CHO treatment. In summary, swimming crabs fed the diet with 1.00% CHO had higher relative levels of volatiles, promoting grassy, fruity, fatty odors in muscle, which may more suit the general public's taste.

Aroma substances are synthesized mainly through the oxidation of fatty acids and the metabolism of amino acids [61]. In the amino acid metabolism pathway, branched-chain amino acids generate branched-chain *α*-keto acid under the action of branched-chain amino acid transaminase (BCAT) [62]. Lipoxygenase (LOX) uses linolenic acid and linoleic acid as precursors to synthesize C6-aldehydes and C6-ethanols, and furthermore, alcohol dehydrogenase (ADH) is involved in the conversion between aldehydes and alcohols [61]. Hence, to explore the volatile production mechanism further, we investigated expression levels of *bcat*, *lox*, and *adh*. The present data showed that the expression level of *bcat* in 1.00% and 2.50% CHO treatments was higher than in 0.12% CHO treatment. However, *lox* and *adh* expression levels were not significantly different among all treatments. This result revealed that CHO could stimulate the biosynthesis of volatile substances by upregulating the expression level of *bcat*. Unfortunately, there are few studies on volatile substances in dietary CHO levels, and further work is needed to explore the underlying mechanisms of these differences.

## 5. Conclusion

In summary, the present study indicated that crabs fed a diet supplemented with CHO (1.00% and 2.50%) could promote the accumulation of n-3 LC-PUFA (especially EPA and DHA) in the hepatopancreas and muscle by activating the gene expression related to LC-PUFA biosynthesis. Furthermore, the results of volatile compounds showed that the muscle of female swimming crabs fed the diet with 1.00% CHO had a more pleasing flavor, which may be due to the upregulated expression level involved in the biosynthesis of volatile substances. However, dietary CHO levels (especially 2.50% CHO level) suppressed amino acid accumulation in the hepatopancreas and muscle by downregulating the TOR pathway and upregulating amino acid catabolism-related genes. Moreover, dietary CHO might regulate nucleotide and FAA metabolism, decreasing the taste and flavor of crabs. Interestingly, dietary CHO had less effect on muscle than on hepatopancreas. Therefore, dietary 1.00% CHO could stimulate the accumulation of lipid nutrition and volatile flavor compounds in the muscle of swimming crab but adverse to the deposition of protein and nonvolatile taste substances in the hepatopancreas.

## Data Availability

The authors confirm that the data supporting the findings of this study are available within the article.

## Supplementary Materials

Supplementary Materials Table S1: formulation and proximate composition of the experimental diets (dry matter basis). Table S2: fatty acid composition (mg/g, dry) of the experimental diets. Table S3: effects of dietary cholesterol level on growth performance and feed utilization (%, wet weight) of female swimming crab (*P. trituberculatus*). Table S4: sodium citrate buffer system compositions for total amino acid analysis. Table S5: HPLC gradient eluent compositions and program for nucleotide analysis. Table S6: primers for real-time quantitative PCR gene expressions of female swimming crab (*P. trituberculatus*). Table S7: effects of dietary cholesterol level on fatty acid composition (mg/g, dry) in hepatopancreas of female swimming crab (*P. trituberculatus*). Table S8: effects of dietary cholesterol level on fatty acid composition (mg/g, dry) in muscle of female swimming crab (*P. trituberculatus*). Table S9: effects of dietary cholesterol level on amino acid compositions (g/100 g dry matter) in hepatopancreas of female swimming crab (*P. trituberculatus*). Table S10: effects of dietary cholesterol level on amino acid compositions (g/100 g dry matter) in muscle of female swimming crab (*P. trituberculatus*). Table S11: effects of dietary cholesterol level on free amino acid (FAA) compositions (mg/g dry matter) in hepatopancreas of female swimming crab (*P. trituberculatus*). Table S12: effects of dietary cholesterol level on the TAV values of free amino acids in hepatopancreas of female swimming crab (*P. trituberculatus*). Table S13: effects of dietary cholesterol level on the TAV values of free amino acids in muscle of female swimming crab (*P. trituberculatus*). Table S14: effects of dietary cholesterol level on free amino acid (FAA) compositions (mg/g dry matter) in muscle of female swimming crab (*P. trituberculatus*). Table S15: effects of dietary cholesterol level on the contents of flavor nucleotides (mg/100 g) in hepatopancreas of female swimming crab (*P. trituberculatus*). Table S16: effects of dietary cholesterol level on the contents of flavor nucleotides (mg/100 g) in muscle of female swimming crab (*P. trituberculatus*). Table S17: effects of dietary cholesterol level on the relative concentrations (ng/g) of volatile compounds in muscle of female swimming crab.

## References

[1] Hardy R. W., Lee C. S. (2010). Aquaculture feed and seafood quality. *Bulletin of Fisheries Research and Development Agency*.

[2] Grigorakis K. (2007). Compositional and organoleptic quality of farmed and wild gilthead sea bream (*Sparus aurata*) and sea bass (*Dicentrarchus labrax*) and factors affecting it: a review. *Aquaculture*.

[3] Tang L., Wang H., Wang C. (2020). Temperature potentially induced distinctive flavor of mud crab *Scylla paramamosain* mediated by gut microbiota. *Scientific Reports*.

[4] Wu X., Zhu S., Zhang H. (2020). Fattening culture improves the gonadal development and nutritional quality of male Chinese mitten crab *Eriocheir sinensis*. *Aquaculture*.

[5] Tu L., Wu X., Wang X., Shi W. (2020). Effects of fish oil replacement by blending vegetable oils in fattening diets on nonvolatile taste substances of swimming crab (*Portunus trituberculatus*). *Journal of Food Biochemistry*.

[6] Mirera D. O., Moksnes P.-O. (2015). Comparative performance of wild juvenile mud crab (*Scylla serrata*) in different culture systems in East Africa: effect of shelter, crab size and stocking density. *Aquaculture International*.

[7] Wu N., Fu X., Zhuang K., Wu X., Wang X. (2019). Effects of dietary replacement of fish oil by vegetable oil on proximate composition and odor profile of hepatopancreas and gonad of Chinese mitten crab (*Eriocheir sinensis*). *Journal of Food Biochemistry*.

[8] Zhou L., Han D., Zhu X., Yang Y., Jin J., Xie S. (2016). Effects of total replacement of fish oil by pork lard or rapeseed oil and recovery by a fish oil finishing diet on growth, health and fish quality of gibel carp (*Carassius auratus gibelio*). *Aquaculture Research*.

[9] Liu Y., Jiao J.-G., Gao S. (2019). Dietary oils modify lipid molecules and nutritional value of fillet in Nile tilapia: a deep lipidomics analysis. *Food Chemistry*.

[10] Zhong W., Zhang S., Li J., Huang W., Wang A. (2011). Effects of dietary replacement of fish oil by conjugated linoleic acid on some meat quality traits of Pacific white shrimp *Litopenaeus vannamei*. *Food Chemistry*.

[11] Kumar V., Sinha A. K., Romano N. (2017). Metabolism and nutritive role of cholesterol in the growth, gonadal development, and reproduction of crustaceans. *Reviews in Fisheries Science & Aquaculture*.

[12] Sheen S.-S. (2000). Dietary cholesterol requirement of juvenile mud crab *Scylla serrata*. *Aquaculture*.

[13] Yepiz-Plascencia G., Vargas-Albores F., Higuera-Ciapara I. (2000). Penaeid shrimp hemolymph lipoproteins. *Aquaculture*.

[14] Grieneisen M. L. (1994). Recent advances in our knowledge of ecdysteroid biosynthesis in insects and crustaceans. *Insect Biochemistry and Molecular Biology*.

[15] Han T., Wang J., Li X. (2015). Effects of dietary cholesterol levels on the growth, molt performance and immunity of juvenile swimming crab, *Portunus trituberculatus*. *Israeli Journal of Aquaculture—Bamidgeh*.

[16] Tian H., Yang C., Yu Y. (2020). Dietary cholesterol level affects growth, molting performance and ecdysteroid signal transduction in *Procambarus clarkii*. *Aquaculture*.

[17] Guo H.-X., Jiang G.-Z., Dai Y.-J. (2022). Effect of dietary cholesterol on growth performance, cholesterol deposition, and lipid metabolism in adult Chinese mitten crab (*eriocheir sinensis*). *Aquaculture Nutrition*.

[18] Guo H., Wang M., Wang X. (2022). Effect of dietary cholesterol on ovarian development of Chinese mitten crabs (*Eriocheir sinensis*). *Frontiers in Marine Science*.

[19] Sun Q., Jiang X., Hou W., He J., Francis D. S., Wu X. (2022). Ovarian fullness affects biochemical composition and nutritional quality of female swimming crab *Portunus trituberculatus*. *Journal of Food Composition and Analysis*.

[20] Fishery Bureau of China Agriculture Department (2023). The China Fishery Statistical Yearbook.

[21] Yuan Y., Wang X., Jin M. (2020). Modification of nutritional values and flavor qualities of muscle of swimming crab (*Portunus trituberculatus*): application of a dietary lipid nutrition strategy. *Food Chemistry*.

[22] Qin K., Li X., Jiang X. (2023). Effect of domestication on nonvolatile flavor substances in mud crabs (*Scylla paramamosain*): a way to improve the quality of commercial crabs. *Journal of Agricultural and Food Chemistry*.

[23] Wang F., Zhu Y., Jiang S., Lin L., Lu J. (2021). Nutritional qualities and sensory characteristics in the hepatopancreas and muscle of female mud crab (*Scylla paramamosain*) in three growth forms: a comparative study. *Lebensmittel–Wissenschaft & Technologie (LWT)*.

[24] Guo Y.-R., Gu S.-Q., Wang X.-C., Zhao L.-M., Zheng J.-Y. (2014). Comparison of fatty acid and amino acid profiles of steamed Chinese mitten crab. *Fisheries Science*.

[25] Mall V., Schieberle P. (2017). Evaluation of key aroma compounds in processed prawns (*Whiteleg Shrimp*) by quantitation and aroma recombination experiments. *Journal of Agricultural and Food Chemistry*.

[26] Yan S., Mao S., Xia Q. (2023). Effects of different habitat space on growth performance and nutritional composition of swimming crabs (*Portunus trituberculatus*). *Aquaculture Research*.

[27] Zhu T., Zhou Q., Yang Z. (2022). Dietary cholesterol promotes growth and ecdysone signalling pathway by modulating cholesterol transport in swimming crabs (*Portunus trituberculatus*). *Animal Nutrition*.

[28] AOAC (2006). Official methods of analysis. *Association of Official Analytical Chemists*.

[29] Wang X., Jin M., Cheng X. (2021). Dietary DHA/EPA ratio affects growth, tissue fatty acid profiles and expression of genes involved in lipid metabolism in mud crab *Scylla paramamosain* supplied with appropriate n-3 LC-PUFA at two lipid levels. *Aquaculture*.

[30] Unnikrishnan U., Paulraj R. (2010). Dietary protein requirement of giant mud crab *Scylla serrata* juveniles fed iso-energetic formulated diets having graded protein levels. *Aquaculture Research*.

[31] Wang S., He Y., Wang Y. (2016). Comparison of flavour qualities of three sourced *Eriocheir sinensis*. *Food Chemistry*.

[32] Song J., Wang H., Wu X., Wang X., Shi W. (2019). The flavor of gonad and meat of female *Portunus trituberculatus* cultured in indoor and outdoor. *Journal of Food Biochemistry*.

[33] Tao H., Du B., Wang H. (2018). Intestinal microbiome affects the distinctive flavor of Chinese mitten crabs in commercial farms. *Aquaculture*.

[34] Luo J., Monroig Ó., Zhou Q. (2021). Environmental salinity and dietary lipid nutrition strategy: effects on flesh quality of the marine euryhaline crab *Scylla paramamosain*. *Food Chemistry*.

[35] Wang X.-X., Yuan Y., Li C.-C. (2020). Partial substitution of fish meal with soy protein concentrate in commercial diets for juvenile swimming crab, *Portunus trituberculatus*. *Animal Feed Science and Technology*.

[36] Livak K. J., Schmittgen T. D. (2001). Analysis of relative gene expression data using real-time quantitative PCR and the 2^−*ΔΔ*CT^ method. *Methods*.

[37] NRC (2011). *Nutrient Requirements of Fish and Shrimp*.

[38] Yan M., Wang W., Huang X., Wang X., Wang Y. (2020). Interactive effects of dietary cholesterol and phospholipids on the growth performance, expression of immune-related genes and resistance against Vibrio alginolyticus in white shrimp (*Litopenaeus vannamei*). *Fish & Shellfish Immunology*.

[39] Harrison K. E. (1990). The role of nutrition in maturation, reproduction and embryonic development of decapod crustacean: a review. *Journal of Shellfish Research*.

[40] Niu J., Chen P.-F., Tian L.-X. (2012). Excess dietary cholesterol may have an adverse effect on growth performance of early post-larval *Litopenaeus vannamei*. *Journal of Animal Science and Biotechnology*.

[41] Gu X., Fu H., Sun S. (2017). Effects of cholesterol on growth, feed utilization, body composition and immune parameters in juvenile oriental river prawn, *Macrobrachium nipponense* (De Haan). *Aquaculture Research*.

[42] Goldberg I. J. (2012). Triglyceride: one molecule at the center of health and disease. *Biochimica et Biophysica Acta (BBA)—Molecular and Cell Biology of Lipids*.

[43] Wu H.-X., Li W.-J., Zhang L. (2022). Microbiota derived butyrate affected the muscle texture of Nile tilapia (*Oreochromis niloticus*) fed with different protein sources. *Food Chemistry*.

[44] Larsen R., Eilertsen K.-E., Elvevoll E. O. (2011). Health benefits of marine foods and ingredients. *Biotechnology Advances*.

[45] Harper C. R., Jacobson T. A. (2005). Usefulness of omega-3 fatty acids and the prevention of coronary heart disease. *American Journal of Cardiology*.

[46] Roynette C. E., Calder P. C., Dupertuis Y. M., Pichard C. (2004). n-3 Polyunsaturated fatty acids and colon cancer prevention. *Clinical Nutrition*.

[47] Sun P., Jin M., Jiao L. (2020). Effects of dietary lipid level on growth, fatty acid profiles, antioxidant capacity and expression of genes involved in lipid metabolism in juvenile swimming crab, *Portunus trituberculatus*. *British Journal of Nutrition*.

[48] Bao Y., Shen Y., Li X. (2022). A new insight into the underlying adaptive strategies of euryhaline marine fish to low salinity environment: through cholesterol nutrition to regulate physiological responses. *Frontiers in Nutrition*.

[49] Bao Y., Shen Y., Wu Z. (2023). High dietary arachidonic acid produces excess eicosanoids, and induces hepatic inflammatory responses, oxidative stress and apoptosis in juvenile *Acanthopagrus schlegelii*. *Aquaculture Reports*.

[50] Monroig O., Tocher D. R., Castro L. F. C., Burdge G. C. (2018). Chapter 3—polyunsaturated fatty acid biosynthesis and metabolism in fish. *Polyunsaturated Fatty Acid Metabolism*.

[51] Vilasoa-Martínez M., López-Hernández J., Lage-Yusty M. A. (2007). Protein and amino acid contents in the crab, *Chionoecetes opilio*. *Food Chemistry*.

[52] Millward D. J. (2012). Identifying recommended dietary allowances for protein and amino acids: a critique of the 2007 WHO/FAO/UNU report. *British Journal of Nutrition*.

[53] FAO/WHO/UNU (1985). *Energy and Protein Requirements. Report of a Joint FAO/WHO/UNU Expert Consultation*.

[54] Hammer V. A., Rogers Q. R., Freedland R. A. (1996). Threonine is catabolized by L-threonine 3-dehydrogenase and threonine dehydratase in hepatocytes from domestic cats (Felis domestica). *The Journal of Nutrition*.

[55] Li R.-X., Chen L.-Y., Limbu S. M. (2023). High cholesterol intake remodels cholesterol turnover and energy homeostasis in Nile tilapia (*Oreochromis niloticus*). *Marine Life Science & Technology*.

[56] Stévant P., Indergård E., Ólafsdóttir A. (2018). Effects of drying on the nutrient content and physico-chemical and sensory characteristics of the edible kelp *Saccharina latissima*. *Journal of Applied Phycology*.

[57] Lu Q., Yu L., Guo X., Wang X. (2023). Volatile compounds, synergistic effects, precursors and impact factors for odor profiles in *Eriocheir sinensis*. *Aquaculture and Fisheries*.

[58] Ran Z., Zhang S., Zhu Y. (2019). Effect of salinity on volatiles in the razor clam investigated by head space-solid phase microextraction/gas chromatography–mass spectrometry. *Fisheries Science*.

[59] Zhuang K., Wu N., Wang X. (2016). Effects of 3 feeding modes on the volatile and nonvolatile compounds in the edible tissues of female Chinese mitten crab (*Eriocheir sinensis*). *Journal of Food Science*.

[60] Bauer K., Garbe D., Surburg H. (2001). Single fragrance and flavor materials. *Common Fragrance and Flavor Materials*.

[61] Zhu X., Li Q., Li J., Luo J., Chen W., Li X. (2018). Comparative study of volatile compounds in the fruit of two banana cultivars at different ripening stages. *Molecules*.

[62] Brosnan J. T., Brosnan M. E. (2006). Branched-chain amino acids: enzyme and substrate regulation. *The Journal of Nutrition*.

