# Supplementary material for: Cholesterol Modifies Nutritional Values and Flavor Qualities in Female Swimming Crab (Portunus trituberculatus)
Source: Aquac Nutr. 2024 Mar 7;2024:7067588. doi: 10.1155/2024/7067588 (PMC11324362; doi:10.1155/2024/7067588)
Supplement: Supplementary Materials — Table S1: formulation and proximate composition of the experimental diets (dry matter basis). Table S2: fatty acid composition (mg/g, dry) of the experimental diets. Table S3: effects of dietary cholesterol level on growth performance and feed utilization (%, wet weight) of female swimming crab (P. trituberculatus). Table S4: sodium citrate buffer system compositions for total amino acid analysis. Table S5: HPLC gradient eluent compositions and program for nucleotide analysis. Table S6: primers for real-time quantitative PCR gene expressions of female swimming crab (P. trituberculatus). Table S7: effects of dietary cholesterol level on fatty acid composition (mg/g, dry) in hepatopancreas of female swimming crab (P. trituberculatus). Table S8: effects of dietary cholesterol level on fatty acid composition (mg/g, dry) in muscle of female swimming crab (P. trituberculatus). Table S9: effects of dietary cholesterol level on amino acid compositions (g/100 g dry matter) in hepatopancreas of female swimming crab (P. trituberculatus). Table S10: effects of dietary cholesterol level on amino acid compositions (g/100 g dry matter) in muscle of female swimming crab (P. trituberculatus). Table S11: effects of dietary cholesterol level on free amino acid (FAA) compositions (mg/g dry matter) in hepatopancreas of female swimming crab (P. trituberculatus). Table S12: effects of dietary cholesterol level on the TAV values of free amino acids in hepatopancreas of female swimming crab (P. trituberculatus). Table S13: effects of dietary cholesterol level on the TAV values of free amino acids in muscle of female swimming crab (P. trituberculatus). Table S14: effects of dietary cholesterol level on free amino acid (FAA) compositions (mg/g dry matter) in muscle of female swimming crab (P. trituberculatus). Table S15: effects of dietary cholesterol level on the contents of flavor nucleotides (mg/100 g) in hepatopancreas of female swimming crab (P. trituberculatus). Table S16: effects of di [file 7067588.f1.docx]

**Supplementary Material**

**Part 1**

Table S1. Formulation and proximate composition of the experimental diets (dry matter basis).

Table S2. Fatty acid composition (mg/g, dry) of the experimental diets.

Table S3. Effects of dietary cholesterol level on growth performance and feed utilization (%, wet weight) of female swimming crab (*Portunus trituberculatus*).

Table S4. Sodium citrate buffer system compositions for total amino acid analysis.

Table S5. HPLC gradient eluent compositions and program for nucleotide analysis.

Table S6. Primers for real-time quantitative PCR gene expressions of female swimming crab (*Portunus trituberculatus*).

Table S7. Effects of dietary cholesterol level on fatty acid composition (mg/g, dry) in hepatopancreas of female swimming crab (*Portunus trituberculatus*).

Table S8. Effects of dietary cholesterol level on fatty acid composition (mg/g, dry) in muscle of female swimming crab (*Portunus trituberculatus*).

Table S9. Effects of dietary cholesterol level on amino acid compositions (g/100 g dry matter) in hepatopancreas of female swimming crab (*Portunus trituberculatus*).

Table S10. Effects of dietary cholesterol level on amino acid compositions (g/100 g dry matter) in muscle of female swimming crab (*Portunus trituberculatus*).

Table S11. Effects of dietary cholesterol level on free amino acid (FAA) compositions (mg/g dry matter) in hepatopancreas of female swimming crab (*Portunus trituberculatus*).

Table S12. Effects of dietary cholesterol level on the TAV values of free amino acids in hepatopancreas of female swimming crab (*Portunus trituberculatus*).

Table S13. Effects of dietary cholesterol level on the TAV values of free amino acids in muscle of female swimming crab (*Portunus trituberculatus*).

Table S14. Effects of dietary cholesterol level on free amino acid (FAA) compositions (mg/g dry matter) in muscle of female swimming crab (*Portunus trituberculatus*).

Table S15. Effects of dietary cholesterol level on the contents of flavor nucleotides (mg/100g) in hepatopancreas of female swimming crab (*Portunus trituberculatus*).

Table S16. Effects of dietary cholesterol level on the contents of flavor nucleotides (mg/100g) in muscle of female swimming crab (*Portunus trituberculatus*).

Table S17. Effects of dietary cholesterol level on the relative concentrations (ng/g) of volatile compounds in muscle of female swimming crab.

**Part 2**

**References**

**Table S1**

Formulation and proximate composition of the experimental diets (dry matter basis).

| Item | Dietary cholesterol levels, % | | |
| --- | --- | --- | --- |
|  | 0.12 | 1.00 | 2.50 |
| Ingredients, % |  |  |  |
| Peru fish meal ^1^ | 25.00 | 25.00 | 25.00 |
| Soybean protein concentrate ^1^ | 7.00 | 7.00 | 7.00 |
| Soybean meal ^1^ | 23.00 | 23.00 | 23.00 |
| Krill meal ^1^ | 3.00 | 3.00 | 3.00 |
| Corn gluten meal ^1^ | 5.00 | 5.00 | 5.00 |
| Wheat flour ^1^ | 23.70 | 23.70 | 23.70 |
| Fish oil ^1^ | 2.00 | 2.00 | 2.00 |
| Palmitic acid ^2^ | 2.00 | 1.25 | 0.00 |
| Cholesterol ^3^ | 0.00 | 0.75 | 2.00 |
| Soybean lecithin ^1^ | 2.00 | 2.00 | 2.00 |
| Vitamin premix ^1^ | 1.00 | 1.00 | 1.00 |
| Mineral premix ^1^ | 2.00 | 2.00 | 2.00 |
| Ca(H_2_PO_4_)_2_ ^1^ | 2.00 | 2.00 | 2.00 |
| Choline chloride ^1^ | 0.30 | 0.30 | 0.30 |
| Sodium alginate ^1^ | 2.00 | 2.00 | 2.00 |
| Total | 100.00 | 100.00 | 100.00 |
| Proximate composition, % |  |  |  |
| Dry matter | 95.46 | 94.85 | 95.45 |
| Crude protein | 46.34 | 46.24 | 46.12 |
| Crude lipid | 8.46 | 8.77 | 8.56 |
| Ash | 11.12 | 10.93 | 10.74 |
| Cholesterol | 0.12 | 1.00 | 2.50 |

^1^ All the ingredients were provided from Ningbo Tech-Bank Feed Co. Ltd., China.

^2^ Palmitic acid: 97% of total fatty acids as palmitic acid methyl ester; Shanghai Yiji Chemical Co., Ltd., China.

^3^ Cholesterol: Shanghai Macklin Biochemical Co., Ltd

**Table S2**

Fatty acid composition (mg/g, dry) of the experimental diets.

| Item | Dietary cholesterol levels, % | | |
| --- | --- | --- | --- |
|  | 0.12 | 1.00 | 2.50 |
| 14: 0 | 0.96 | 1.05 | 1.06 |
| 16: 0 | 12.65 | 10.27 | 5.60 |
| 18: 0 | 1.16 | 1.24 | 1.15 |
| 20: 0 | 0.08 | 0.09 | 0.08 |
| ΣSFA | 14.86 | 12.65 | 7.89 |
| 16: 1n | 0.90 | 1.05 | 1.02 |
| 18: 1n - 9 | 5.87 | 6.40 | 6.06 |
| 20: 1n - 9 | 0.23 | 0.20 | 0.20 |
| 22: 1n - 11 | 0.06 | 0.04 | 0.02 |
| ΣMUFA | 7.06 | 7.69 | 7.29 |
| 18: 2n - 6 | 2.93 | 3.07 | 3.07 |
| 18: 3n - 6 | 0.01 | 0.02 | 0.02 |
| 20: 2n - 6 | 0.03 | 0.03 | 0.03 |
| 20: 4n - 6 (ARA) | 0.13 | 0.16 | 0.16 |
| Σn - 6PUFA | 3.11 | 3.28 | 3.27 |
| 18: 3n - 3 | 0.72 | 0.83 | 0.77 |
| 18: 4n - 3 | 0.47 | 0.49 | 0.45 |
| 20: 4n - 3 | 0.16 | 0.19 | 0.17 |
| 20: 5n - 3 (EPA) | 0.89 | 1.15 | 1.18 |
| 22: 5n - 3 | 0.29 | 0.44 | 0.46 |
| 22: 6n - 3 (DHA) | 2.49 | 2.54 | 2.57 |
| Σn - 3PUFA | 5.01 | 5.65 | 5.59 |
| n - 3/n - 6PUFA | 1.61 | 1.72 | 1.71 |
| n - 3LC - PUFA | 3.83 | 4.33 | 4.37 |
| DHA/EPA | 2.80 | 2.21 | 2.19 |

SFA, saturated fatty acids; MUFA, monounsaturated fatty acids; n - 6 PUFA, omega 6 polyunsaturated fatty acids; n - 3PUFA, omega 3 polyunsaturated fatty acids; n - 3/n - 6 PUFA, omega 3 polyunsaturated fatty acids : omega 6 polyunsaturated fatty acids; n - 3 LC-PUFA, omega 3 long chain polyunsaturated fatty acids; DHA/EPA, 22:6n - 3/20:5n - 3.

**Table S3**

Effects of dietary cholesterol level on growth performance and feed utilization (%, wet weight) of female swimming crab (*Portunus trituberculatus*).

| Item | Dietary cholesterol level, % | | |
| --- | --- | --- | --- |
|  | 0.12 | 1.00 | 2.50 |
| IBW, g | 1.50 ± 0.06 | 1.52 ± 0.05 | 1.50 ± 0.12 |
| FBW, g | 94.20 ± 3.63^b^ | 118.30 ± 6.53^a^ | 117.00 ± 4.80^a^ |
| PWG, % | 5852.57 ± 128.15^b^ | 7549.06 ± 394.03^a^ | 7256.96 ± 20.83^a^ |
| SGR, %/day | 3.07 ± 0.02^b^ | 3.26 ± 0.04^a^ | 3.20 ± 0.02^a^ |
| FCR | 2.14 ± 0.07^a^ | 1.84 ± 0.08^b^ | 1.66 ± 0.01^b^ |
| Survival, % | 75.00 ± 0.00 | 75.00 ± 0.00 | 83.33 ± 5.89 |
| MR | 5.17 ± 0.07^c^ | 6.32 ± 0.14^a^ | 5.86 ± 0.06^b^ |

IBW, initial body weight; FBW, final body weight; PWG, Percent weight gain; SGR, Specific growth rate; FCR, feed conversion ratio; MR, molting ratio.

^a - c^ Values in the same line with different superscripts are significantly different (*P* ˂0.05). Data are reported as the mean and SEM (*n* = 4).

Table S4

Sodium citrate buffer system compositions for total amino acid analysis.

| Item | PH-1 | PH-2 | PH-3 | PH-4 | PH-5 |
| --- | --- | --- | --- | --- | --- |
| The concentration of sodium (N) | 0.16 | 0.20 | 0.20 | 1.20 | 0.20 |
| Density (g/cm^3^) | 1.02 | 1.02 | 1.02 | 1.06 | 1.00 |
| PH | 3.30 | 3.20 | 4.00 | 4.90 | - |
| Distillated water (ml, nearly) | 700.00 | 700.00 | 700.00 | 700.00 | 700.00 |
| Trisodium citrate dehydrate (g) | 6.19 | 7.74 | 13.31 | 26.67 | - |
| Sodium hydroxide (g) | - | - | - | - | 8.00 |
| Sodium chloride (g) | 5.66 | 7.07 | 3.74 | 54.35 | - |
| Citric acid monohydrate (g) | 19.80 | 22.00 | 12.80 | 6.10 | - |
| Alcohol (ml) | 130.00 | 20.00 | 4.00 | - | 100.00 |
| Benzyl alcohol (ml) | - | - | - | 5.00 | - |
| Dihydroxyethyl sulfide (ml) | 5.00 | 5.00 | 5.00 | - | - |
| Polyethylen (ml) | 4.00 | 4.00 | 4.00 | 4.00 | 4.00 |
| Octanoic acid (ml) | 0.10 | 0.10 | 0.10 | 0.10 | 0.10 |

Table S5

HPLC gradient eluent compositions and program for nucleotide analysis.

| Item | Mobile phase A (%) | Mobile phase B (%) |
| --- | --- | --- |
| Composition |  |  |
| Methanol | 50 ml | 800 ml |
| Water | 950 ml | 200 ml |
| Phosphorous acid | 0.5 ml | 0.5 ml |
| Time/min |  |  |
| 0 min | 100 | 0 |
| 5 min | 100 | 0 |
| 15 min | 90 | 10 |
| 25 min | 0 | 100 |
| 30 min | 0 | 100 |
| 35 min | 100 | 0 |
| 40 min | 100 | 0 |

**Table S6**

Primers for real - time quantitative PCR gene expressions of female swimming crab (*Portunus trituberculatus*).

| Gene | Nucleotide sequence (5’ – 3’) | Size, bp | | GenBank reference or Publication |
| --- | --- | --- | --- | --- |
| *fad2* | F: GCAGTGAGAGACAGGACGGA | | 241bp | PRJNA432636 |
|  | R: CTGGATGGTTAGGGTTTGGG | |  |  |
| *elovl* | F: TGTATCTGTACTACCTGCTGG | | 100bp | PRJNA432636 |
|  | R: GCTGTGAACTGTATCATCTGA | |  |  |
| *elovl4* | F: AGCTACACAGGATGAAGGACC | | 215bp | PRJNA432636 |
|  | R: GAGCAGCATAATGGCAAGG | |  |  |
| *ast* | F: TTTACAACGAGCGCATTGGT | | 206bp | PRJNA428031 |
|  | R: GATGCGTCCACTCATTGTCC | |  |  |
| *alt1* | F: AGGTCCTGTCACTTTGCTCA | | 186bp | PRJNA428031 |
|  | R: GGCCATCCCTTTGCTGTATG | |  |  |
| *tdh* | F: CATCTCTGCTGACACCATGC | | 250bp | PRJNA428031 |
|  | R: CCTCACAGCTGCCACAATTT | |  |  |
| *s6* | F: CACTAACAACCGTGTGCGAC | | 148bp | Wang et al., 2019 |
|  | R: ACCCTTCTTGATGATGACCAG | |  |  |
| *akt* | F: GGACTACGAGGCACCAAGAA | | 179bp | Wang et al., 2019 |
|  | R: TGGACCACTTCATCACGCTC | |  |  |
| *s6k1* | F: CGCCCCTCAGATTTCCAGT | | 175bp | Wang et al., 2019 |
|  | R: TCTCAGCCTTTGTGTGCG | |  |  |
| *tor* | F: TGTGGACATAGGGCAAACTG | | 174bp | Wang et al., 2019 |
|  | R: GACCGCTTCACCAAATCATC | |  |  |
| *4e-bp1* | F: GGCTGAACTTCCAAATGACT | | 142bp | Wang et al., 2019 |
|  | R: TTCTTGGGTGGGGTCTTG | |  |  |
| *eif4e1a* | F: TGAACAAGCAGCAGCGAG | | 114bp | Wang et al., 2019 |
|  | R: TGACCACAGCACCACACA | |  |  |
| *eif4e2* | F: GCTTTCAGGAGGACATCATC | | 144bp | Wang et al., 2019 |
|  | R: TGAGGGAGTCATTGTGAGTCT | |  |  |
| *eif4e3* | F: CCCTGGACCTTCTGGATTGA | | 181bp | Wang et al., 2019 |
|  | R: TCCTGTTACCCCTCATCAAG | |  |  |
| *lox* | F: GGTGTGCTAGACCCAGATGT | | 228bp | PRJNA428031 |
|  | R: GCCTTCCTCATCATTGCCAG | |  |  |
| *adh* | F: GTGTCTGCCAGAAATGGGTG | | 215bp | PRJNA428031 |
|  | R: CATCTGACCCAGCAAAAGCA | |  |  |
| *bcat* | F: GATAGACTTTTGCCGGCCAG | | 208bp | PRJNA428031 |
|  | R: CATGCCTTCAAACAGCTCCA | |  |  |
| *β-actin* | F: CGAAACCTTCAACACTCCCG | | - | FJ641977.1 |
|  | R:GGATAGCGTGAGGAAGGGCATA | |  | |

*fad2*, fatty acyl desaturase 2; elovl, elongase; elovl4, elongase 4; *ast*, aminotransferase; *alt1*, alanine aminotransferase 1; *tdh*, L-threonine 3-dehydrogenase; *s6*, ribosomal protein S6; *akt*, protein kinases B; *s6k1*, ribosomal protein S6 kinase1; *tor*, target of rapamycin; *4e-bp1*, eukaryotic initiation factor 4E binding protein-1; *eif4e1a*, eukaryotic translation initiation factor *4E-1A*; *eif4e2*, eukaryotic translation initiationfactor 4E-2; *eif4e3*, eukaryotic translation initiation factor 4E-3; *lox*, lipoxygenase; *adh*, alcohol dehydrogenase; *bcat*, branched-chain amino acid transaminase.

**Table S7**

Effects of dietary cholesterol level on fatty acid composition (mg/g, dry) in hepatopancreas of female swimming crab (*Portunus trituberculatus*).

| Item | Dietary cholesterol levels, % | | | |
| --- | --- | --- | --- | --- |
|  | 0.12 | 1.00 | 2.50 |  |
| 14: 0 | 2.91 ± 0.06b | 3.36 ± 0.13ab | 3.74 ± 0.16a |  |
| 16: 0 | 32.57 ± 1.20a | 32.61 ± 0.84a | 28.45 ± 0.58b |  |
| 18: 0 | 9.15 ± 0.01b | 9.61 ± 0.02a | 8.73 ± 0.02c |  |
| 20: 0 | 0.67 ± 0.02 | 0.67 ± 0.04 | 0.66 ± 0.02 |  |
| ΣSFA | 45.31 ± 1.16ab | 46.23 ± 0.87a | 41.58 ± 0.73b |  |
| 16: 1n | 5.33 ± 0.12b | 6.10 ± 0.03ab | 6.32 ± 0.36a |  |
| 18: 1n - 9 | 38.31 ± 0.09c | 43.30 ± 0.79b | 46.49 ± 0.53a |  |
| 20: 1n - 9 | 2.43 ± 0.02b | 2.37 ± 0.01b | 2.66 ± 0.05a |  |
| 22: 1n - 11 | 0.14 ± 0.02b | 0.24 ± 0.02a | 0.18 ± 0.01ab |  |
| ΣMUFA | 46.22 ± 0.21c | 52.00 ± 0.82b | 55.64 ± 0.85a |  |
| 18: 2n - 6 | 7.84 ± 0.15b | 8.88 ± 0.31ab | 9.62 ± 0.45a |  |
| 18: 3n - 6 | 0.03 ± 0.00b | 0.19 ± 0.09ab | 0.37 ± 0.01a |  |
| 20: 2n - 6 | 2.31 ± 0.03c | 2.61 ± 0.05b | 3.53 ± 0.02a |  |
| 20: 4n - 6 (ARA) | 0.64 ± 0.06b | 0.89 ± 0.02a | 0.86 ± 0.01a |  |
| 22: 4n - 6 | 0.09 ± 0.01b | 0.14 ± 0.00a | 0.15 ± 0.00a |  |
| Σn - 6PUFA | 10.92 ± 0.25c | 12.70 ± 0.43b | 14.52 ± 0.47a |  |
| 18: 3n - 3 | 1.30 ± 0.02 | 1.34 ± 0.11 | 1.47 ± 0.09 |  |
| 18: 4n - 3 | 0.65 ± 0.01 | 0.58 ± 0.06 | 0.56 ± 0.08 |  |
| 20: 4n - 3 | 0.53 ± 0.03 | 0.58 ± 0.03 | 0.63 ± 0.04 |  |
| 20: 5n - 3 (EPA) | 3.05 ± 0.04b | 3.70 ± 0.22a | 3.74 ± 0.07a |  |
| 22:6n - 3 (DHA) | 8.87 ± 0.11b | 9.40 ± 0.20ab | 9.78 ± 0.07a |  |
| Σn - 3PUFA | 15.75 ± 0.08b | 17.49 ± 0.59ab | 18.25 ± 0.41a |  |
| n - 3/n - 6PUFA | 1.44 ± 0.03a | 1.38 ± 0.03a | 1.26 ± 0.02b |  |
| n - 3LC - PUFA | 13.80 ± 0.08^b^ | 15.56 ± 0.44a | 16.22 ± 0.25^a^ |  |
| n - 6LC - PUFA | 3.05 ± 0.10^c^ | 3.63 ± 0.06^b^ | 4.53 ± 0.02^a^ |  |
| DHA/EPA | 2.91 ± 0.08 | 2.56 ± 0.13 | 2.61 ± 0.04 |  |

SFA, saturated fatty acids; MUFA, monounsaturated fatty acids; n-6 PUFA, n-6 polyunsaturated fatty acids; n-3 PUFA, n - 3 polyunsaturated fatty acids; n-3 LC-PUFA, n-3 long chain polyunsaturated fatty acids; DHA/EPA, 22:6n−3/20:5n−3.

^a - c^ Values in the same line with different superscripts are significantly different (*P* ˂0.05). Data are reported as the mean and SEM (*n* = 4).

**Table S8**

Effects of dietary cholesterol level on fatty acid composition (mg/g, dry) in muscle of female swimming crab (*Portunus trituberculatus*).

| Item | Dietary cholesterol levels, % | | | |
| --- | --- | --- | --- | --- |
|  | 0.12 | 1.00 | 2.50 |  |
| 14: 0 | 0.10 ± 0.01b | 0.24 ± 0.04a | 0.18 ± 0.01ab |  |
| 16: 0 | 4.74 ± 0.11a | 4.85 ± 0.09a | 4.32 ± 0.02b |  |
| 18: 0 | 1.95 ± 0.06b | 2.12 ± 0.01a | 2.27 ± 0.09a |  |
| 20: 0 | 0.05 ± 0.01 | 0.06 ± 0.00 | 0.05 ± 0.00 |  |
| ΣSFA | 6.81 ± 0.11 | 7.27 ± 0.14 | 6.81 ± 0.14 |  |
| 16: 1n | 0.27 ± 0.03b | 0.59 ± 0.07a | 0.36 ± 0.03b |  |
| 18: 1n - 9 | 4.70 ± 0.06b | 5.79 ± 0.24a | 4.98 ± 0.14b |  |
| 20: 1n - 9 | 0.07 ± 0.00 | 0.10 ± 0.02 | 0.07 ± 0.00 |  |
| ΣMUFA | 5.04 ± 0.09b | 6.48 ± 0.32a | 5.41 ± 0.17b |  |
| 18: 2n - 6 | 1.35 ± 0.02b | 1.59 ± 0.04a | 1.58 ± 0.03a |  |
| 20: 2n - 6 | 0.24 ± 0.01b | 0.30 ± 0.02ab | 0.32 ± 0.01a |  |
| 20: 4n - 6 (ARA) | 0.21 ± 0.01b | 0.27 ± 0.00a | 0.25 ± 0.01a |  |
| Σn - 6PUFA | 1.84 ± 0.02b | 2.17 ± 0.06a | 2.15 ± 0.06a |  |
| 18: 3n - 3 | 0.11 ± 0.00b | 0.14 ± 0.01a | 0.14 ± 0.00a |  |
| 20: 4n - 3 | 0.04 ± 0.00b | 0.06 ± 0.03a | 0.04 ± 0.00b |  |
| 20: 5n - 3 (EPA) | 3.29 ± 0.12 | 3.57 ± 0.13 | 3.77 ± 0.15 |  |
| 22: 5n - 3 | 0.10 ± 0.01 | 0.13 ± 0.02 | 0.11 ± 0.01 |  |
| 22: 6n - 3 (DHA) | 3.04 ± 0.03b | 3.44 ± 0.03a | 3.33 ± 0.09a |  |
| Σn - 3PUFA | 6.57 ± 0.08b | 7.33 ± 0.08a | 7.39 ± 0.14a |  |
| n - 3/n - 6PUFA | 3.57 ± 0.02 | 3.39 ± 0.13 | 3.44 ± 0.04 |  |
| n - 3LC - PUFA | 6.46 ± 0.08b | 7.20±0.09a | 7.25 ± 0.14a |  |
| n - 6LC - PUFA | 0.45 ± 0.01b | 0.57 ± 0.02a | 0.57 ± 0.02a |  |
| DHA/EPA | 0.93 ± 0.04 | 0.97 ± 0.05 | 0.89 ± 0.05 |  |

SFA, saturated fatty acids; MUFA, monounsaturated fatty acids; n-6 PUFA, n-6 polyunsaturated fatty acids; n-3 PUFA, n-3 polyunsaturated fatty acids; n-3 LC-PUFA, n - 3 long chain polyunsaturated fatty acids; DHA/EPA, 22:6n-3/20:5n-3.

^a - c^ Values in the same line with different superscripts are significantly different (*P* ˂0.05). Data are reported as the mean and SEM (*n* = 4).

**Table S9**

Effects of dietary cholesterol level on amino acid compositions (g/100 g dry matter) in hepatopancreas of female swimming crab (*Portunus trituberculatus*).

| Item | Dietary cholesterol levels, % | | | |
| --- | --- | --- | --- | --- |
|  | 0.12 | 1.00 | 2.50 |  |
| Thr | 1.65 ± 0.05a | 1.43 ± 0.05b | 1.34 ± 0.02b |  |
| Val | 1.76 ± 0.04a | 1.58 ± 0.04b | 1.49 ± 0.04b |  |
| Met | 0.82 ± 0.03a | 0.70 ± 0.04b | 0.67 ± 0.01b |  |
| Ile | 1.27 ± 0.04a | 1.14 ± 0.04b | 1.08 ± 0.02b |  |
| Leu | 2.40 ± 0.07a | 2.15 ± 0.08ab | 2.03 ± 0.05b |  |
| Phe | 1.46 ± 0.04a | 1.32 ± 0.06ab | 1.23 ± 0.01b |  |
| Lys | 2.43 ± 0.08a | 2.16 ± 0.10ab | 1.99 ± 0.05b |  |
| His | 0.93 ± 0.02a | 0.86 ± 0.02b | 0.80 ± 0.01b |  |
| Arg | 2.03 ± 0.04a | 1.90 ± 0.08ab | 1.75 ± 0.08b |  |
| EAA | 14.77 ± 0.41a | 13.25 ± 0.44b | 12.38 ± 0.27b |  |
| Asp | 3.13 ± 0.10a | 2.70 ± 0.09b | 2.56 ± 0.02b |  |
| Ser | 1.49 ± 0.05a | 1.28 ± 0.05b | 1.21 ± 0.03b |  |
| Glu | 4.23 ± 0.13a | 3.67 ± 0.14b | 3.44 ± 0.10b |  |
| Gly | 1.34 ± 0.04a | 1.22 ± 0.04ab | 1.16 ± 0.02b |  |
| Ala | 1.46 ± 0.05a | 1.24 ± 0.05ab | 1.13 ± 0.12b |  |
| Cys | 0.85 ± 0.03a | 0.69 ± 0.02b | 0.63 ± 0.03b |  |
| Tyr | 1.34 ± 0.04a | 1.16 ± 0.05b | 1.10 ± 0.01b |  |
| Pro | 2.09 ± 0.0.22a | 1.50 ± 0.05b | 1.35 ± 0.05b |  |
| NEAA | 16.65 ± 0.54a | 13.46 ± 0.46b | 12.57 ± 0.35b |  |
| FLAA | 10.44 ± 0.15^a^ | 8.83 ± 0.31^b^ | 8.28 ± 0.26^b^ |  |
| TAA | 31.79 ± 0.67a | 26.71 ± 0.89b | 24.96 ± 0.62b |  |
| EAA/TAA | 0.48 ± 0.01b | 0.50 ± 0.00a | 0.50 ± 0.00a |  |

EAA, essential amino acids; NEAA, nonessential amino acids; FLAA, flavor amino acids including aspartic acid, glutamic acid, glycine, and alanine; TAA, summed of total amino acids; EAA/TAA, the ratio of essential amino acids to total amino acids.

^a - c^ Values in the same line with different superscripts are significantly different (*P* ˂0.05). Data are reported as the mean and SEM (*n* = 4).

**Table S10**

Effects of dietary cholesterol level on amino acid compositions (g/100 g dry matter) in muscle of female swimming crab (*Portunus trituberculatus*).

| Item | Dietary cholesterol levels, % | | | |
| --- | --- | --- | --- | --- |
|  | 0.12 | 1.00 | 2.50 |  |
| Thr | 3.01 ± 0.03a | 3.00 ± 0.03a | 2.85 ± 0.02b |  |
| Val | 3.26 ± 0.02a | 3.25 ± 0.02a | 3.05 ± 0.05b |  |
| Met | 1.81 ± 0.03 | 1.74 ± 0.09 | 1.60 ± 0.04 |  |
| Ile | 2.96 ± 0.02a | 2.93 ± 0.05a | 2.75 ± 0.01b |  |
| Leu | 5.48 ± 0.04a | 5.46 ± 0.05a | 5.13 ± 0.05b |  |
| Phe | 2.78 ± 0.01a | 2.77 ± 0.03a | 2.60 ± 0.05b |  |
| Lys | 5.83 ± 0.13a | 5.75 ± 0.14ab | 5.37 ± 0.05b |  |
| His | 1.46 ± 0.02 | 1.44 ± 0.02 | 1.40 ± 0.02 |  |
| Arg | 8.73 ± 0.08 | 8.79 ± 0.03 | 8.41 ± 0.11 |  |
| EAA | 35.31 ± 0.21a | 35.14 ± 0.40a | 33.40 ± 0.28b |  |
| Asp | 6.27 ± 0.05a | 6.18 ± 0.02a | 5.95 ± 0.03b |  |
| Ser | 2.80 ± 0.04a | 2.80 ± 0.02a | 2.65 ± 0.03b |  |
| Glu | 10.74 ± 0.06 | 10.74 ± 0.20 | 10.30 ± 0.05 |  |
| Gly | 5.26 ± 0.36 | 5.32 ± 0.12 | 4.90 ± 0.12 |  |
| Ala | 3.65 ± 0.08a | 3.73 ± 0.01a | 3.34 ± 0.08b |  |
| Cys | 1.04 ± 0.03a | 1.00 ± 0.01a | 0.91 ± 0.03b |  |
| Tyr | 2.45 ± 0.03 | 2.43 ± 0.05 | 2.35 ± 0.01 |  |
| Pro | 7.35 ± 0.07 | 7.41 ± 0.19 | 8.09 ± 0.75 |  |
| NEAA | 39.55 ± 0.66 | 39.63 ± 0.41 | 38.49 ± 0.85 |  |
| FLAA | 25.92 ± 0.52a | 25.98 ± 0.16a | 24.36 ± 0.09b |  |
| TAA | 74.86 ± 0.86a | 74.77 ± 0.81a | 71.79 ± 0.26b |  |
| EAA/TAA | 0.47 ± 0.00 | 0.47 ± 0.00 | 0.46 ± 0.00 |  |

EAA, essential amino acids; NEAA, nonessential amino acids; FLAA, flavor amino acids including aspartic acid, glutamic acid, glycine, and alanine; TAA, summed of total amino acids; EAA/TAA, the ratio of essential amino acids to total amino acids.

^a - c^ Values in the same line with different superscripts are significantly different (*P* ˂0.05). Data are reported as the mean and SEM (*n* = 4).

**Table S11**

Effects of dietary cholesterol level on free amino acid (FAA) compositions (mg/g dry matter) in hepatopancreas of female swimming crab (*Portunus trituberculatus*).

| Item | Taste threshold, mg/g | | Dietary cholesterol levels, % | | |
| --- | --- | --- | --- | --- | --- |
|  |  |  | 0.12 | 1.00 | 2.50 |
| Ala | | 0.60 | 3.51 ± 0.25^a^ | 3.03 ± 0.04^a^ | 2.04 ± 0.05^b^ |
| Gly | | 1.30 | 4.56 ± 0.14^a^ | 4.45 ± 0.02^ab^ | 4.15 ± 0.04^b^ |
| Ser | | 1.50 | 0.28 ± 0.04 | 0.26 ± 0.01 | 0.26 ± 0.02 |
| Thr | | 2.60 | 1.56 ± 0.15^a^ | 1.49 ± 0.02^a^ | 0.78 ± 0.04^b^ |
| Pro | | 3.00 | 2.79 ± 0.00^a^ | 2.47 ± 0.09^a^ | 1.88 ± 0.13^b^ |
| Sweet FAA | | - | 12.71 ± 0.57^a^ | 11.57 ± 0.14^a^ | 9.26 ± 0.09^b^ |
| Glu | | 0.30 | 5.12 ± 0.20^a^ | 4.10 ± 0.02^b^ | 2.33 ± 0.15^c^ |
| Asp | | 1.00 | 1.21 ± 0.00^a^ | 0.91 ± 0.04^b^ | 0.43 ± 0.04^c^ |
| Umami FAA | | - | 6.34 ± 0.20^a^ | 5.00 ± 0.05^b^ | 2.76 ± 0.12^c^ |
| Val | | 0.40 | 2.08 ± 0.17^a^ | 1.53 ± 0.10^b^ | 1.00 ± 0.04^c^ |
| Met | | 0.30 | 1.97 ± 0.06^a^ | 1.46 ± 0.06^b^ | 1.16 ± 0.00^c^ |
| Leu | | 1.90 | 4.63 ± 0.16^a^ | 3.43 ± 0.08^b^ | 2.84 ± 0.07^c^ |
| Ile | | 0.90 | 1.94 ± 0.09^a^ | 1.33 ± 0.05^b^ | 0.93 ± 0.02^c^ |
| Phe | | 0.90 | 4.03 ± 0.01^a^ | 2.65 ± 0.10^b^ | 1.91 ± 0.07^c^ |
| His | | 0.20 | 0.80 ± 0.12^a^ | 0.70 ± 0.07^ab^ | 0.36 ± 0.02^b^ |
| Arg | | 0.50 | 8.57 ± 0.48^a^ | 7.06 ± 0.10^b^ | 5.61 ± 0.24^c^ |
| Lys | | 0.50 | 5.24 ± 0.29^a^ | 4.45 ± 0.07^a^ | 3.62 ± 0.06^b^ |
| Trp | | - | 1.53 ± 0.03^a^ | 1.06 ± 0.07^b^ | 0.74 ± 0.08^c^ |
| Tyr | | - | 5.15 ± 0.17^a^ | 3.30 ± 0.22^b^ | 2.34 ± 0.14^c^ |
| Bitter FAA | | - | 35.94 ± 1.17^a^ | 27.07 ± 0.78^b^ | 20.52 ± 0.25^c^ |
| Tau | | - | 2.59 ± 0.37 | 2.01 ± 0.22 | 2.69 ± 0.23 |
| CYS - S | | - | 1.08 ± 0.04^a^ | 0.81 ± 0.05^b^ | 0.32 ± 0.02^c^ |
| Asn | | - | 0.71 ± 0.13 | 0.66 ± 0.07 | 0.39 ± 0.02 |
| Gln | | - | 1.18 ± 0.05 | 1.11 ± 0.04 | 1.07 ± 0.15 |
| Other FAA | | - | 5.55 ± 0.39 | 4.58 ± 0.10 | 4.47 ± 0.33 |
| Tatal FAA | | - | 60.53 ± 1.67^a^ | 48.36 ± 0.73^b^ | 36.86 ± 0.42^c^ |

Sweet FAA, total sweet free amino acids; Umami FAA, total umami free amino acids; Bitter FAA, total bitter free amino acids; Other FAA, total other free amino acids; TFAA, total free amino acids.

^a - c^ Values in the same line with different superscripts are significantly different (*P* ˂0.05). Data are reported as the mean and SEM (*n* = 4).

**Table S12**

Effects of dietary cholesterol level on the TAV values of free amino acids in hepatopancreas of female swimming crab (*Portunus trituberculatus*).

| Item | Taste threshold, mg/g | Dietary cholesterol levels, % | | |
| --- | --- | --- | --- | --- |
|  |  | 0.12 | 1.00 | 2.50 |
| Ala | 0.60 | 5.85 ± 0.42^a^ | 5.05 ± 0.06^a^ | 3.41 ± 0.09^b^ |
| Gly | 1.30 | 3.51 ± 0.11^a^ | 3.42 ± 0.02^ab^ | 3.19 ± 0.03^b^ |
| Ser | 1.50 | 0.18 ± 0.03 | 0.17 ± 0.01 | 0.17 ± 0.02 |
| Thr | 2.60 | 0.60 ± 0.06^a^ | 0.57 ± 0.01^a^ | 0.30 ± 0.01^b^ |
| Pro | 3.00 | 0.93 ± 0.00^a^ | 0.82 ± 0.03^a^ | 0.63 ± 0.04^b^ |
| Glu | 0.30 | 17.08 ± 0.68^a^ | 13.66 ± 0.04^b^ | 7.76 ± 0.50^c^ |
| Asp | 1.00 | 1.21 ± 0.00^a^ | 0.91 ± 0.04^b^ | 0.43 ± 0.04^c^ |
| Val | 0.40 | 5.21 ± 0.43^a^ | 3.82 ± 0.25^b^ | 2.52 ± 0.09^c^ |
| Met | 0.30 | 6.55 ± 0.22^a^ | 4.85 ± 0.21^b^ | 3.86 ± 0.01^c^ |
| Leu | 1.90 | 2.44 ± 0.09^a^ | 1.81 ± 0.04^b^ | 1.49 ± 0.04^c^ |
| Ile | 0.90 | 2.16 ± 0.10^a^ | 1.48 ± 0.05^b^ | 1.04 ± 0.02^c^ |
| Phe | 0.90 | 4.48 ± 0.01^a^ | 2.95 ± 0.11^b^ | 2.12 ± 0.07^c^ |
| His | 0.20 | 4.02 ± 0.59^a^ | 3.48 ± 0.34^ab^ | 1.81 ± 0.12^b^ |
| Arg | 0.50 | 17.13 ± 0.97^a^ | 14.12 ± 0.20^b^ | 11.22 ± 0.49^c^ |
| Lys | 0.50 | 10.47 ± 0.57^a^ | 9.10 ± 0.13^a^ | 7.24 ± 0.12^b^ |

^a - c^ Values in the same line with different superscripts are significantly different (*P* ˂0.05). Data are reported as the mean and SEM (*n* = 4).

**Table S13**

Effects of dietary cholesterol level on the TAV values of free amino acids in muscle of female swimming crab (*Portunus trituberculatus*).

| Item | Taste threshold, mg/g | Dietary cholesterol levels, % | | | |
| --- | --- | --- | --- | --- | --- |
|  |  | 0.12 | 1.00 | 2.50 |  |
| Ala | 0.60 | 3.42 ± 0.17^a^ | 3.40 ± 0.15^a^ | 2.23 ± 0.19^b^ |  |
| Gly | 1.30 | 18.00 ± 0.19^a^ | 16.93 ± 0.69^a^ | 13.49 ± 0.24^b^ |  |
| Ser | 1.50 | 0.11 ± 0.01^a^ | 0.10 ± 0.00^ab^ | 0.09 ± 0.00^b^ |  |
| Thr | 2.60 | 0.09 ± 0.01 | 0.09 ± 0.00 | 0.09 ± 0.00 |  |
| Pro | 3.00 | 4.31 ± 0.06^a^ | 4.29 ± 0.17^a^ | 3.73 ± 0.09^b^ |  |
| Glu | 0.30 | 4.32 ± 0.17^a^ | 3.82 ± 0.13^a^ | 2.96 ± 0.22^b^ |  |
| Asp | 1.00 | 0.25 ± 0.01^a^ | 0.17 ± 0.01^b^ | 0.11 ± 0.01^b^ |  |
| Val | 0.40 | 0.86 ± 0.08^a^ | 0.77 ± 0.01^ab^ | 0.60 ± 0.02^b^ |  |
| Met | 0.30 | 4.50 ± 0.04^a^ | 4.24 ± 0.41^a^ | 2.62 ± 0.19^b^ |  |
| Leu | 1.90 | 0.30 ± 0.04^a^ | 0.26 ± 0.01^ab^ | 0.18 ± 0.01^b^ |  |
| Ile | 0.90 | 0.20 ± 0.01^a^ | 0.20 ± 0.00^a^ | 0.16 ± 0.00^b^ |  |
| Phe | 0.90 | 0.31 ± 0.04^a^ | 0.25 ± 0.01^ab^ | 0.19 ± 0.01^b^ |  |
| His | 0.20 | 0.71 ± 0.06 | 0.60 ± 0.00 | 0.61 ± 0.04 |  |
| Arg | 0.50 | 33.98 ± 0.39^a^ | 33.52 ± 1.68^a^ | 25.58 ± 0.32^b^ |  |
| Lys | 0.50 | 1.40 ± 0.19 | 1.39 ± 0.17 | 1.01 ± 0.02 |  |

^a - c^ Values in the same line with different superscripts are significantly different (*P* ˂0.05). Data are reported as the mean and SEM (*n* = 4).

**Table S14**

Effects of dietary cholesterol level on free amino acid (FAA) compositions (mg/g dry matter) in muscle of female swimming crab (*Portunus trituberculatus*).

| Item | Taste threshold, mg/g | | Dietary cholesterol levels, % | | | |
| --- | --- | --- | --- | --- | --- | --- |
|  |  |  | 0.12 | 1.00 | 2.50 |  |
| Ala | | 0.60 | 2.05 ± 0.06^a^ | 2.04 ± 0.09^a^ | 1.34 ± 0.11^b^ |  |
| Gly | | 1.30 | 23.40 ± 0.25^a^ | 22.01 ± 0.90^a^ | 17.53 ± 0.32^b^ |  |
| Ser | | 1.50 | 0.16 ± 0.01^a^ | 0.14 ± 0.01^ab^ | 0.13 ± 0.00^b^ |  |
| Thr | | 2.60 | 0.24 ± 0.02 | 0.24 ± 0.01 | 0.24 ± 0.00 |  |
| Pro | | 3.00 | 12.92 ± 0.19^a^ | 12.88 ± 0.50^a^ | 11.18 ± 0.28^b^ |  |
| Sweet FAA | | - | 38.77 ± 0.26^a^ | 37.32 ± 0.32^a^ | 30.43 ± 0.61^b^ |  |
| Glu | | 0.30 | 1.30 ± 0.05^a^ | 1.14 ± 0.04^a^ | 0.89 ± 0.06^b^ |  |
| Asp | | 1.00 | 0.25 ± 0.01^a^ | 0.17 ± 0.01^b^ | 0.11 ± 0.01^b^ |  |
| Umami FAA | | - | 1.55 ± 0.06^a^ | 1.31 ± 0.04^b^ | 1.00 ± 0.06^c^ |  |
| Val | | 0.40 | 0.34 ± 0.03^a^ | 0.31 ± 0.00^ab^ | 0.24 ± 0.01^b^ |  |
| Met | | 0.30 | 1.35 ± 0.01^a^ | 1.27 ± 0.12^a^ | 0.79 ± 0.06^b^ |  |
| Leu | | 1.90 | 0.56 ± 0.07^a^ | 0.49 ± 0.02^ab^ | 0.35 ± 0.02^b^ |  |
| Ile | | 0.90 | 0.18 ± 0.01^a^ | 0.18 ± 0.00^a^ | 0.14 ± 0.00^b^ |  |
| Phe | | 0.90 | 0.28 ± 0.04^a^ | 0.22 ± 0.01^ab^ | 0.17 ± 0.01^b^ |  |
| His | | 0.20 | 0.14 ± 0.01 | 0.12 ± 0.00 | 0.12 ± 0.01 |  |
| Arg | | 0.50 | 16.99 ± 0.20^a^ | 16.76 ± 0.84^a^ | 12.79 ± 0.16^b^ |  |
| Lys | | 0.50 | 0.70 ± 0.09 | 0.69 ± 0.09 | 0.50 ± 0.01 |  |
| Trp | | - | 0.03 ± 0.00 | 0.07 ± 0.01 | 0.06 ± 0.00 |  |
| Tyr | | - | 0.32 ± 0.07 | 0.31 ± 0.01 | 0.21 ± 0.01 |  |
| Bitter FAA | | - | 20.90 ± 0.53^a^ | 20.43 ± 1.05^a^ | 15.37 ± 0.17^b^ |  |
| Tau | | - | 3.32 ± 0.10^a^ | 3.19 ± 0.01^a^ | 1.89 ± 0.16^b^ |  |
| Cys-s | | - | 0.52 ± 0.01 | 0.53 ± 0.01 | 0.55 ± 0.01 |  |
| Asn | | - | 0.16 ± 0.03 | 0.12 ± 0.02 | 0.12 ± 0.01 |  |
| Gln | | - | 1.81 ± 0.36 | 1.83 ± 0.38 | 1.51 ± 0.23 |  |
| Other FAA | | - | 5.81 ± 0.28^a^ | 5.67 ± 0.39^a^ | 4.07 ± 0.20^b^ |  |
| Tatal FAA | | - | 67.03 ± 0.99^a^ | 64.74 ± 0.79^a^ | 50.87 ± 0.42^b^ |  |

Sweet FAA, total sweet free amino acids; Umami FAA, total umami free amino acids; Bitter FAA, total bitter free amino acids; Other FAA, total other free amino acids; TFAA, total free amino acids.

^a - c^ Values in the same line with different superscripts are significantly different (*P* ˂0.05). Data are reported as the mean and SEM (*n* = 4).

**Table S15**

Effects of dietary cholesterol level on the contents of flavor nucleotides (mg/100g) in hepatopancreas of female swimming crab (*Portunus trituberculatus*).

| Item | Taste threshold, mg/100g | Dietary cholesterol levels, % | | |
| --- | --- | --- | --- | --- |
|  |  | 0.12 | 1.00 | 2.50 |
| AMP | 50.00 | 43.49 ± 5.22^a^ | 26.76 ± 1.51^b^ | 6.55 ± 1.51^c^ |
| GMP | 12.50 | 19.79 ± 0.56^a^ | 14.14 ± 1.38^b^ | 4.71 ± 0.27^c^ |
| IMP | 25.00 | 32.45 ± 4.70 | 29.45 ± 1.85 | 30.67 ± 1.34 |

AMP, Adenosine monophosphate; GMP, Guanosine Monophosphate; IMP, inosine monophosphate. ^a - c^ Values in the same line with different superscripts are significantly different (*P* ˂0.05). Data are reported as the mean and SEM (*n* = 4).

**Table S16**

Effects of dietary cholesterol level on the contents of flavor nucleotides (mg/100g) in muscle of female swimming crab (*Portunus trituberculatus*).

| Item | Taste threshold, mg/100g | Dietary cholesterol levels, % | | |
| --- | --- | --- | --- | --- |
|  |  | 0.12 | 1.00 | 2.50 |
| AMP | 50.00 | 130.14 ± 2.50 | 126.56 ± 2.16 | 124.68 ± 1.96 |
| GMP | 12.50 | 14.59 ± 1.89^a^ | 12.53 ± 0.38^ab^ | 8.99 ± 0.71^b^ |
| IMP | 25.00 | 26.20 ± 1.88 | 26.36 ± 3.79 | 27.27 ± 3.95 |

AMP, Adenosine monophosphate; GMP, Guanosine Monophosphate; IMP, inosine monophosphate. ^a - c^ Values in the same line with different superscripts are significantly different (*P* ˂0.05). Data are reported as the mean and SEM (*n* = 4).

**Table S17**

Effects of dietary cholesterol level on the relative concentrations (ng/g) of volatile compounds in muscle of female swimming crab.

| Item | Dietary cholesterol levels, % | | |
| --- | --- | --- | --- |
|  | 0.12 | 1.00 | 2.50 |
| Cyclobutane, 1,1,2,3,3 -pentamethyl - | - | 1.11 ± 0.08 | - |
| Spiro[2,4] hepta - 4,6 - diene | - | - | 13.83 ± 1.00 |
| Styrene | 3.16 ± 0.15^b^ | 9.15 ± 0.13^a^ | 9.89 ± 0.34^a^ |
| Bicyclo[3.1.0]hex - 2 - ene, 2 -methyl - 5 - (1 - methylethyl) - | 3.84 ± 0.09^a^ | 1.06 ± 0.06^b^ | - |
| Heptane, 2,2,4,6,6-pentamethyl - | 8.15 ± 0.72^c^ | 13.32 ± 0.18^a^ | 10.15 ± 0.30^b^ |
| D - Limonene | 8.79 ± 0.46^a^ | 3.27 ± 0.47^b^ | 2.44 ± 0.40^b^ |
| Hydrocarbons | 23.94 ± 0.53^c^ | 27.91 ± 0.52^b^ | 36.30 ± 1.06^a^ |
| 4,6 - Heptadiyn - 3 - one | - | 7.81 ± 0.42^b^ | 12.31 ± 0.33^a^ |
| 5 - Hepten - 2 - one, 6 - methyl - | - | 1.96 ± 0.12 | 0.92 ± 0.02 |
| n - Octyl phenyl ketone | 2.65 ± 0.28 | - | - |
| Benz[b]1,4 - oxazin - 3(2H) - one, 4 - benzoyl - | 1.54 ± 0.00^a^ | 1.05 ± 0.01^b^ | - |
| Acetophenone | - | 1.47 ± 0.16 | - |
| Ketones | 4.19 ± 0.28^b^ | 12.29 ± 0.31^a^ | 13.23 ± 0.32^a^ |
| Isophthalic acid, di(2 -methoxyethyl) ester | 2.90 ± 0.44 | - | - |
| Benzoic acid, 2 - ethylhexyl ester | 3.00 ± 0.53 | - | - |
| 2,5 - Pyrrolidinedione, 1 -(benzoyloxy) - | - | - | 2.46 ± 0.51 |
| Chloroacetic acid, heptyl ester | - | - | 4.75 ± 0.18 |
| Propanoic acid, 2 - methyl -, 3 -hydroxy - 2,2,4 - trimethylpentyl ester | 25.38 ± 1.74 | - | - |
| Esters | 31.28 ± 1.69^a^ | - | 7.21 ± 0.53^b^ |
| Cyclohexanol, 2 - methyl -, cis - | - | 2.95 ± 0.01 | - |
| 1 - Octen - 3 - ol | - | 3.83 ± 0.34^a^ | 1.84 ± 0.17^b^ |
| (S) - (+) - 5 - Methyl - 1 -heptanol | - | 1.78 ± 0.17 | - |
| Alcohols | - | 8.56 ± 0.34^a^ | 1.84 ± 0.17^b^ |
| 3 - Cyclohexene - 1 -acetaldehyde, .alpha.,4 – dimethyl - | 2.71 ± 0.03 | - | - |
| Benzaldehyde | - | 3.33 ± 0.20 | - |
| Nonanal | - | 18.00 ± 0.29 | - |
| Aldehydes | 2.71 ± 0.03^b^ | 21.33 ± 0.25^a^ | - |
| Arsine | 30.17 ± 0.02^c^ | 57.01 ± 0.27^b^ | 64.43 ± 0.32^a^ |
| Trichloromethane | 21.69 ± 0.26^a^ | 13.54 ± 1.89^b^ | 18.45 ± 2.28^ab^ |
| Benzenemethanol, .alpha. - [1 -(ethylmethylamino)ethyl] - | - | - | 3.33 ± 0.72 |
| 4 - Ethylbenzamide | 11.13 ± 2.31^b^ | 22.60 ± 2.11^a^ | 9.27 ± 1.67^b^ |
| Toluene | 9.89 ± 0.65^b^ | 20.91 ± 1.63^a^ | 14.25 ± 1.43^b^ |
| Oxime -, methoxy – phenyl - | - | 2.51 ± 0.26 | - |
| Ethylbenzene | 5.98 ± 0.49 | 8.94 ± 1.50 | 6.69 ± 0.62 |
| Benzene, 1,3 – dimethyl - | 15.43 ± 1.64 | 18.40 ± 1.68 | 13.30 ± 1.21 |
| Furan, 2 - pentyl - | - | 2.95 ± 0.12 | - |
| 2 - Azido - 2,4,4,6,6 - pentamethyl heptane | 1.79 ± 0.32 | - | - |
| Dicyclopentadiene diepoxide | 1.70 ± 0.02 | - | - |
| Naphthalene | 1.83 ± 0.21^b^ | 5.69 ± 0.80^a^ | 4.18 ± 0.49^a^ |
| 2H - Imidazole - 2 - thione, 1,3 -dihydro - 1 - methyl- | 9.44 ± 0.42 | - | - |
| Naphthalene, 2 - methyl - | 8.18 ± 0.27^a^ | 7.87 ± 0.30^a^ | 5.60 ± 0.44^b^ |
| 1H - Imidazole, 1 - (1 -naphthalenylmethyl) - | - | 4.82 ± 0.17 | - |
| Others | 117.24 ± 2.96^b^ | 165.25 ± 7.39^a^ | 139.51 ± 6.32^b^ |

^a -c^ Values in the same line with different superscripts are significantly different (*P* ˂0.05). Data are reported as the mean and SEM (*n* = 4).

**References**

Wang X X, Yuan Y, Li C C, Zhou F, Jin M, Sun P, et al. (2020). Partial substitution of fish meal with soy protein concentrate in commercial diets for juvenile swimming crab, *Portunus trituberculatus*. *Animal Feed Science and Technology*, *259*, 114290. <https://doi.org/10.1016/j.anifeedsci.2019.114290>
